# Discovering, Integrating, and Reinterpreting the Molecular Logic of Life: From Classical Theories of Heredity to an Extended Functional Perspective on the Central Dogma

**DOI:** 10.3390/life16010079

**Published:** 2026-01-04

**Authors:** Andrei Cristian Grădinaru

**Affiliations:** “Ion Ionescu de la Brad” Iasi University of Life Sciences, 3 Sadoveanu Alley, 700490 Iasi, Romania; andrei.gradinaru@iuls.ro; Tel.: +40-726104403

**Keywords:** Central Dogma, epigenetics, heredity, life molecules, molecular evolution

## Abstract

The conceptual understanding of genetic information has evolved from early philosophical speculation to the molecular precision of contemporary biology. Initial debates over the nature of heredity, including Mendel’s hereditary factors and the longstanding protein versus nucleic acid controversy, underscored the difficulty of identifying the true substrate of inheritance. Subsequent discoveries, including reverse transcription, protein-based infectivity (prions), transposable elements, and the regulatory functions of non-coding RNAs, revealed molecular processes that operate at the boundaries of, or alongside, Crick’s original formulation of the Central Dogma of Molecular Biology. Importantly, these findings do not violate the directional rules of information transfer defined by the Central Dogma (DNA → RNA → protein), but instead reshape how, when, and under what constraints these canonical flows are executed in living systems. Epigenetic and epigenetic-like mechanisms, including DNA methylation, histone modifications, chromatin topology, non-canonical DNA conformations, and cytoplasmic inheritance, introduce regulatory layers that modulate information flow without constituting independent information matrices. In parallel, genome innovation, through *de novo* gene birth, and genome erosion, through pseudogenization, demonstrate that the repertoire of DNA → RNA → protein pathways is itself evolutionarily dynamic. This narrative integrative review reconstructs the historical milestones that culminated in the Central Dogma and synthesizes subsequent discoveries that expand its functional realization. By revisiting the Central Dogma through an extended, holistic lens, this article argues that DNA, RNA, and proteins function not only as carriers of genetic information, but also as active participants in its regulation, contextualization, and evolutionary diversification, without departing from the core directional principles originally articulated by Crick. For reader convenience, a dedicated section entitled “Abbreviations and Key Molecular Terms” is provided at the end of the manuscript to facilitate navigation and interdisciplinary accessibility.

## 1. Introduction

The quest to understand how life perpetuates itself across generations has accompanied biology since its earliest stages. Long before the molecular era, debates between preformationism and epigenesis shaped biological thought throughout the seventeenth and eighteenth centuries [1,2,3,4]. The nineteenth century introduced transformative conceptual frameworks, including Lamarck’s proposal of the inheritance of acquired characteristics [5,6], Darwin’s theory of evolution by natural selection [7] and his concept of pangenesis [8,9,10,11], and Mendel’s demonstration of particulate inheritance [12]. Weismann’s germ plasm theory subsequently established a foundational separation between hereditary material and somatic influence, providing a critical conceptual bridge toward modern genetics [13]. The discovery of chromosomes [14], the elucidation of mitosis and meiosis by Mayzel, Bütschli, Strasburger, and Flemming [15,16], and the rediscovery of Mendel’s work at the turn of the twentieth century [17], together with the chromosomal theory of heredity [18,19,20], transformed heredity into a mechanistic science. The identification of DNA as the carrier of genetic information [21] and the description of its double-helical structure [22] established a molecular foundation for biological inheritance.

Within this context, Francis Crick’s Central Dogma of Molecular Biology, articulated in 1958 and refined in 1970 [23,24], provided a unifying logical framework for the directional flow of genetic information from DNA to RNA to protein. Subsequent discoveries revealed that this canonical information flow operates within a broader and more versatile molecular context than originally envisioned. Reverse transcription [25,26], RNA catalysis [27,28], protein-based information transfer in prions [29], and mobile genetic elements [30] introduced additional pathways that expanded the operational scope of the dogma without violating its directional principles. In parallel, the rise of epigenetics revealed multiple layers of heritable regulation that act independently of primary DNA sequence. DNA methylation [31], histone modifications [32], non-coding RNAs [33,34,35,36,37,38,39,40,41,42,43,44,45,46,47,48], and chromatin remodeling mechanisms exposed a multi-layered regulatory architecture that interacts with and modulates the canonical DNA → RNA → protein pathway. Moreover, non-canonical DNA conformations, such as A- and Z-DNA [49,50,51], demonstrate that genetic information can also be encoded in higher-order structural states, adding an epigenetic-like dimension to gene regulation. Cytoplasmic inheritance, particularly through mitochondria in animal cell, further illustrates how non-nuclear genetic systems can influence nuclear gene expression via metabolic and signaling pathways, constituting an additional regulatory layer with epigenetic-like consequences. Contemporary perspectives on the Central Dogma also incorporate the dynamic evolution of genomes themselves. Processes such as *de novo* gene birth, gene diversification, and pseudogenization emphasize that genomes are not static repositories of information, but adaptive systems in which genetic content is continuously generated, repurposed, or silenced over evolutionary time.

This review aims to trace the intellectual lineage culminating in the Central Dogma of Molecular Biology through a narrative, integrative approach that synthesizes historical, theoretical, and contemporary molecular findings. Importantly, the present work does not propose new primary information flows, nor does it challenge the directional constraints articulated by Crick. Instead, it examines how a growing array of molecular mechanisms, including epigenetic, epitranscriptomic, structural, cytoplasmic, and RNA-centered processes, functionally shape, constrain, and contextualize canonical information transfer in living systems. These mechanisms modulate whether genetic information is expressed, silenced, buffered, amplified, or transmitted across cellular and generational timescales, without constituting independent informational matrices equivalent to DNA, RNA, or protein.

Throughout this review, a clear distinction is made between mechanisms that constitute autonomous, heritable information carriers and those that modulate the expression, stability, or contextual deployment of genetic information. The latter are referred to as “epigenetic-like” processes and are not proposed to represent independent hereditary matrices or to violate the directional constraints of the Central Dogma. For clarity, Table 1 summarizes the principal molecular mechanisms discussed in this review, indicating their molecular substrate, mode of action, and whether they constitute stable heritable systems or epigenetic-like regulatory processes.

## 2. Heredity Beginnings: From Archaic Theories to Hereditary Factors

Looking back in time, defining heredity and further establishing its mechanisms was a great challenge for scientists and philosophers alike. From early beliefs in the spontaneous generation of organisms to more structured developmental and evolutionary theories, scientists progressively built the conceptual foundations of inheritance. The Preformist views (De Graaf, Malpighi, Swammerdam, Leeuwenhoek, Bonnet), the Epigenesis theory (Harvey, Wolff), and the early evolutionary ideas of Lamarck and Darwin, all shaped the first scientific attempts in early understanding the process of heredity.

A major conceptual leap occurred with Mendel’s Laws of Heredity, which provided the first mechanism-based and predictive framework for character inheritance, introducing “hereditary factors” as discrete units. Shortly after, Darwin proposed the Pangenesis theory as a transitional explanation integrating acquired traits into heredity.

**Figure 1:**
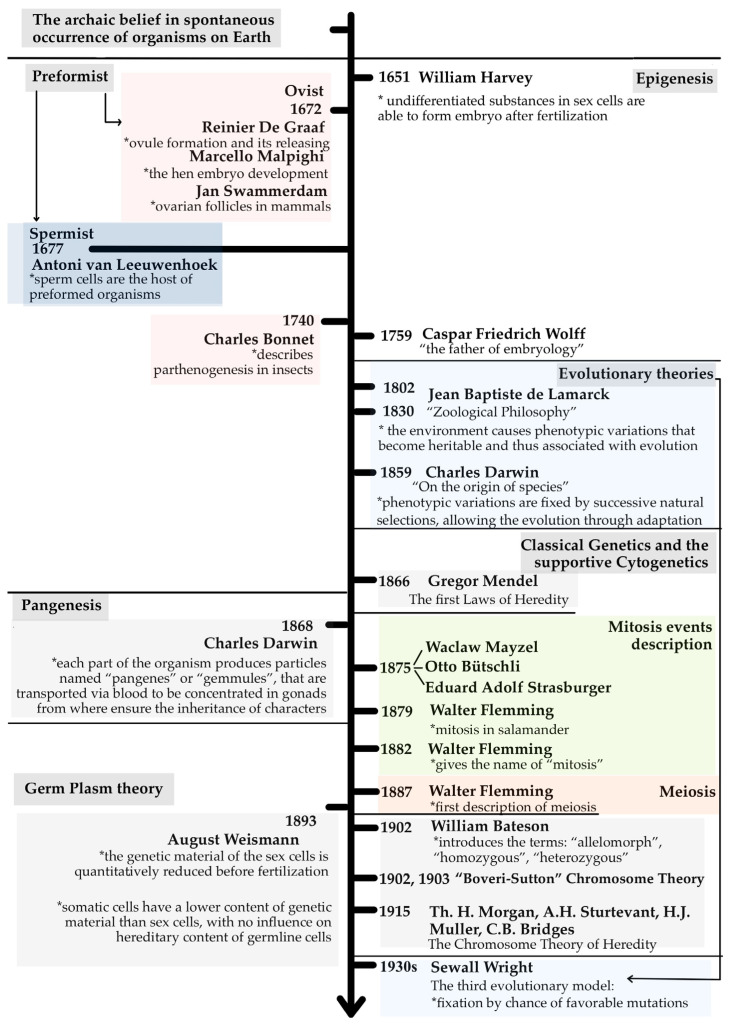
Timeline of Heredity Research: From Pre-Scientific Ideas to the Cytological Substrate (original).

The first cytological evidence of mitosis and meiosis began to appear (Mayzel, Bütschli, Strasburger, Flemming), as well as proofs of genetic material finding in the structure of sperm and ovum, its prior quantitative reduction before fertilization, and the presence of “determinants” (today’s “genes”) in the structure of germ cell’s nucleus, as Weismann proposed in the Germ Plasm theory (Figure 1).

### 2.1. From Tiny Humans to Gradual Growth: Understanding Early Development Theories

Compared with earlier beliefs in the spontaneous appearance of life, the Preformist and Epigenesis theories of the 17th century represented substantial advances in under-standing organismal development and inheritance.

The spread of the optical microscope, first manufactured between 1595 and 1609 by Zacharias Janssen and his father Johannes Sachariasssen, alias Hans [52], initiated a new era of biological observation. Its initial ~10× magnification [53] was soon improved to 25–250× through the independent work of Robert Hooke and Antoni van Leeuwenhoek. Hooke provided the first documented description of a microorganism (namely *Mucor microfungus*, in 1665) [54,55], whereas Leeuwenhoek, in 1677, reported numerous microscopic protozoa and bacteria [55,56].

Leeuwenhoek’s pioneering observations on spermatozoa contributed decisively to the Spermist (Animalculist) branch of Preformism, proposing that spermatozoa contain miniature preformed organisms that unfold during development [1]. Spermism was favored partly due to the accessibility of semen for microscopic examination. In contrast, Ovism, supported by evidence of ovule formation and release provided by Reinier De Graaf in 1672 [1,57,58], assigned the preformed embryo to the egg. Ovism gained momentum through the embryological studies of Marcello Malpighi (hen embryos development, 1672), those of Swammerdam (who described ovarian follicles in mammals in the same year as Reinier De Graaf, 1672), and those of Charles Bonnet, who describes in 1740 the phenomenon of parthenogenesis in insects, whereby the embryo develops from an unfertilized egg [1]. Despite sharing the core idea of miniature organisms pre-existing within gametes, Preformist theories denied the joint contribution of sperm and ovum. This mutual exclusion was reinforced by Leeuwenhoek’s dismissal of the ovum’s developmental role and, conversely, by Karl Ernst von Baer’s belief that spermatozoa were mere parasites [2].

In contrast, the Epigenesis theory, emerging in the same period, challenged the pre-formed-organism view. It proposed that embryos develop gradually from an initially unstructured zygote through sequential differentiation [3]. William Harvey, in 1651, suggested that undifferentiated substances within sex cells could give rise to the embryo after fertilization [2,59]. The theory was solidified by Caspar Friedrich Wolff, who, in 1759, demonstrated through detailed studies of chick embryos that tissues and organs emerge progressively from simple to complex, and distributed over a series of distinct systems (nervous, muscular, digestive, etc.) [1,4]. Wolff’s work marked a pivotal shift, introducing a dynamic and developmental perspective on life that laid the foundations of modern embryology and developmental biology.

### 2.2. Heredity in Evolution

Evolutionary theory has fundamentally shaped the modern understanding of heredity by linking inheritance to variation, adaptation, and long-term population dynamics. From its earliest formulations, evolutionary thinking has sought to explain not only how biological traits change over time, but also how such changes are transmitted across generations.

*Jean-Baptiste Lamarck, in Philosophie Zoologique (1802; 1830)* [5,6], proposed one of the first explicit evolutionary mechanisms by arguing that environmental conditions generate new functional needs, which in turn induce phenotypic modifications that are inherited by offspring. This model of the inheritance of acquired characteristics established an early conceptual link between adaptation and heredity [60]. Although subsequently rejected as a general mechanism of evolution, Lamarck’s framework was historically important in highlighting the role of environmental influences in shaping biological form.

*Charles Darwin’s On the Origin of Species (1859)* [7] fundamentally reframed evolutionary change as the outcome of natural selection acting on heritable variation [61]. In Darwin’s model, variation arises without foresight, and environmental pressures selectively favor those variants that confer reproductive advantage, leading to adaptive evolution over time [62]. However, Darwin lacked a mechanistic understanding of heredity, which prompted him to propose provisional hypotheses, such as Pangenesis, in an attempt to explain the transmission of traits across generations.

*The modern evolutionary synthesis* reconciled evolutionary theory with Mendelian genetics, firmly establishing mutation, recombination, and segregation as the primary sources of heritable variation. Subsequent theoretical developments further refined this framework. In particular, *Sewall Wright’s shifting balance theory*, developed in the 1930s [63,64], emphasized the combined roles of natural selection, genetic drift, and population structure, demonstrating that allele frequencies can change not only through adaptive selection but also through stochastic processes, especially in subdivided populations [65]. In this respect, Wright extended Darwin’s framework by showing that beneficial variants may become fixed by chance as well as by selection. Together, Darwinian selection and Wrightian population genetics shaped a coherent evolutionary–genetic framework that underpins modern evolutionary biology.

In recent decades, advances in molecular biology have expanded the range of mechanisms known to influence heritable variation, including epigenetic regulation, transposable element activity, and large-scale regulatory reorganization. These discoveries have occasionally been interpreted as lending support to neo-Lamarckian ideas. For example, Skinner’s neo-Lamarckian/neo-Darwinian reconciliation [60] proposes that environmental factors can induce epigenetic modifications, that such modifications may influence DNA sequence over time, and that the resulting molecular changes can generate heritable phenotypic variation subject to natural selection. However, as emphasized by Brosius (2005), genome-level processes such as transposable element mobilization, regulatory rewiring, and epigenetic modulation generate contingent, non-teleological variation rather than adaptive instruction [66]. These mechanisms introduce stochastic and context-dependent molecular changes that increase genomic diversity and regulatory plasticity. Although such changes may subsequently be co-opted by natural selection, they do not encode environmentally directed or purposeful genetic outcomes. Accordingly, environmentally responsive molecular processes do not constitute a revival of classical Lamarckian inheritance of acquired characteristics. Instead, they expand the sources of evolutionary novelty by enhancing genomic flexibility and regulatory complexity, while remaining fully compatible with a Darwinian framework in which natural selection acts as a filter on variation after it arises. In this view, heredity in evolution reflects a dynamic interplay between random molecular events, regulatory plasticity, population-level processes, and selective forces, rather than a directed transmission of adaptive responses.

### 2.3. The Genetic Journey: From Mendel’s Laws to Cellular Division and Chromosomes

Darwin revolutionized biology by proposing natural selection as the engine of evolution, yet he lacked a clear hereditary mechanism. Mendel’s work, published in 1866, provided exactly what Darwin’s theory needed: a predictable system of inheritance based on discrete hereditary units.

Working with pea plants, Mendel identified segregation, dominance, and independent assortment, naming the units of inheritance “hereditary factors” and inferring their paired nature (corresponding to modern genotypes) [12]. This was remarkable given that chromosomes had not yet been linked to heredity. The chromosomal basis of inheritance was established only in 1902–1903 with the Boveri-Sutton Chromosome Theory [18,19,20]. Later, Thomas Hunt Morgan and colleagues (A.H. Sturtevant, H.J. Muller and C.B. Bridges) provided evidence for gene linkage, recombination, and chromosomal mapping in 1915 [67]. Bateson introduced the terms “allele”, “homozygous”, and “heterozygous” in 1902; Johannsen first introduced the concepts of “gene”, “genotype”, and “phenotype” in 1905 and subsequently formalized and rigorously defined them in 1909, emphasizing the conceptual distinction between genes and observable characteristics [53].

Mendel also anticipated key aspects of meiosis, such as allele segregation and gamete formation, decades before the process was described. Early observations of mitosis were made by Wacław Mayzel (in studies on frog corneal epithelium), Otto Bütschli (in other animal species), and Eduard Adolf Strasburger (in plants) in 1875 [15], followed by Flemming’s detailed description and naming of mitosis (1879, 1882) [16]. Fertilization, although known, was still subject to controversy. Jean-Louis Prévost and Jean Baptiste André Dumas (1824) demonstrated sperm entry into the ovum in the amphibian *Rana temporaria*; Gustave-Adolphe Thuret (1854) captured gamete fusion in algae belonging to the genus *Fucus*. Yet some scientists, including Th.W. Bischoff (1842) and J.P. Müller (1844), remained skeptical about the major role that the sperm plays in this process [1].

Mendel’s results, strikingly consistent and mathematically precise, later sparked de-bates regarding selective reporting or limited cross types [68,69,70]. Despite criticism, Mendel’s methodological rigor and quantitative interpretation allowed him to uncover essential principles of heredity long before the cellular and molecular mechanisms were known.

## 3. From Particles to Lineage: Pangenesis and Germ Plasm Theories

During the mid and late 19th century, the question of how hereditary information is transmitted became embedded in two influential and ideologically distinct frameworks: Darwin’s Pangenesis Theory and Weismann’s Germ Plasm Theory. These contrasted views illustrate the transition from speculative particle-based concepts of heredity to line-age-based models grounded in cytology and early genetics.

Charles Darwin, inspired in part by ideas attributed to Hippocrates, proposed the Theory of Pangenesis in 1868 as a mechanism of somatic-to-germline inheritance. He envisioned that all tissues of the body release minute particulate entities (“gemmules”) that circulate throughout the organism, accumulate in the gonads, and transmit both inherited and acquired traits to the next generation [8,9,10,11]. In Darwin’s view, these gemmules carried information reflective of the organism’s physiological state and environmental experience, conceptually aligning with Lamarckian ideas on the inheritance of acquired characteristics [60]. The particulate nature of these hypothetical hereditary units was later refined and explicitly formalized by Hugo de Vries, who introduced the term “pangens” in 1889, attempting to reconcile Darwinian inheritance with emerging cytological observations [71].

However, Pangenesis quickly encountered empirical challenges. Francis Galton’s blood transfusion experiments in differently pigmented rabbits failed to support the transfer of heritable traits through circulating particles [8,72], casting doubt on the theory. A more definitive conceptual break came with August Weismann’s Germ Plasm Theory, formally presented in his German monograph (1892) and subsequently disseminated through later editions and translations (1893) [13]. This framework reoriented heredity as a strictly unidirectional process, flowing from germ cells to somatic cells, but never in the reverse direction.

Weismann proposed that hereditary information resides exclusively in the nucleus of germ cells, which contain a complete and continuous lineage of “determinants” (the predecessors of modern “genes”). He recognized key principles that would later be confirmed cytological: (*i*) the localization of hereditary material in the nuclei of sperm and ova; (*ii*) the reduction in hereditary content prior to fertilization (i.e., meiotic reduction); (*iii*) the transmission of determinants through the germline. Conversely, Weismann assigned the “somatoplasm” (plasma of somatic cells) with a limited and derivative set of determinants and denied any capacity of somatic tissues to alter or regenerate the germline. As a consequence, the development of germline cells from somatic cells was deemed impossible, and acquired characteristics were considered non-heritable. After fertilization, the germ plasm was thought to guide embryonic development through its division and distribution into nascent somatic tissues, providing each lineage with the determinants required for differentiation [73,74].

In recent decades, several assumptions underlying both theories have undergone significant revision. While Darwin’s original “gemmules” were metaphorical, modern findings suggest that certain circulating molecular entities, such as extracellular nucleic acids (including cell-free fetal DNA), small RNAs carried by sperm, and even prion-like agents in plant sap or animal blood, may participate in intercellular or intergenerational communication, conceptually echoing aspects of Pangenesis [72]. Similarly, the strict Weismann barrier, which posits a one-way information flow from germline to soma, has been softened by discoveries showing that somatic cells can influence germline epigenetic states. Somatic regulatory cells, such as Sertoli cells, can modulate patterns of DNA demethylation and remethylation during male germ cell development, thereby contributing to epigenetic variability that may affect offspring phenotype [73,74]. Moreover, the early assumption that somatic cells lack the full complement of hereditary determinants has been refuted. Modern developmental biology reveals that early embryonic blastomeres (derived from the zygote after fertilization) possess totipotency or pluripotency, enabling them to differentiate into both somatic and germline precursors. This finding reverses the hierarchical separation imagined by Weismann and demonstrates that, in higher organisms, somatic lineages originate from cells initially capable of giving rise to germline tissue. Together, the reinterpretation of these classical theories reveals a more dynamic, multilayered view of heredity, one that includes not only DNA sequence but also epigenetic mechanisms, intercellular signaling, and context-dependent regulation. These modern perspectives prepare the conceptual ground for understanding the later expansion of the Central Dogma of molecular biology.

## 4. The Substrate Dilemma of Heredity: DNA or Proteins?

Advances in molecular biology and genetics now allow us to view hereditary information as flowing through multiple molecular pathways, extending far beyond the early assumption that DNA is the sole substrate of heredity. This broadened perspective aligns with the expanded version of the Central Dogma of Molecular Biology, articulated by Francis Crick in 1970 [24], which revised his initial 1958 formulation of unidirectional information transfer from DNA to protein, with RNA as an intermediate molecule [23].

Crick’s 1970 revision [24] provided a more flexible conceptual framework in which: (*i*) DNA, while the principal hereditary substrate, is not the only possible carrier of genetic information; (*ii*) RNA can act in additional informational transfers, including reverse flow processes; (*iii*) protein-to-protein information transfer, although considered highly im-probable, is not theoretically excluded.

**Figure 2:**
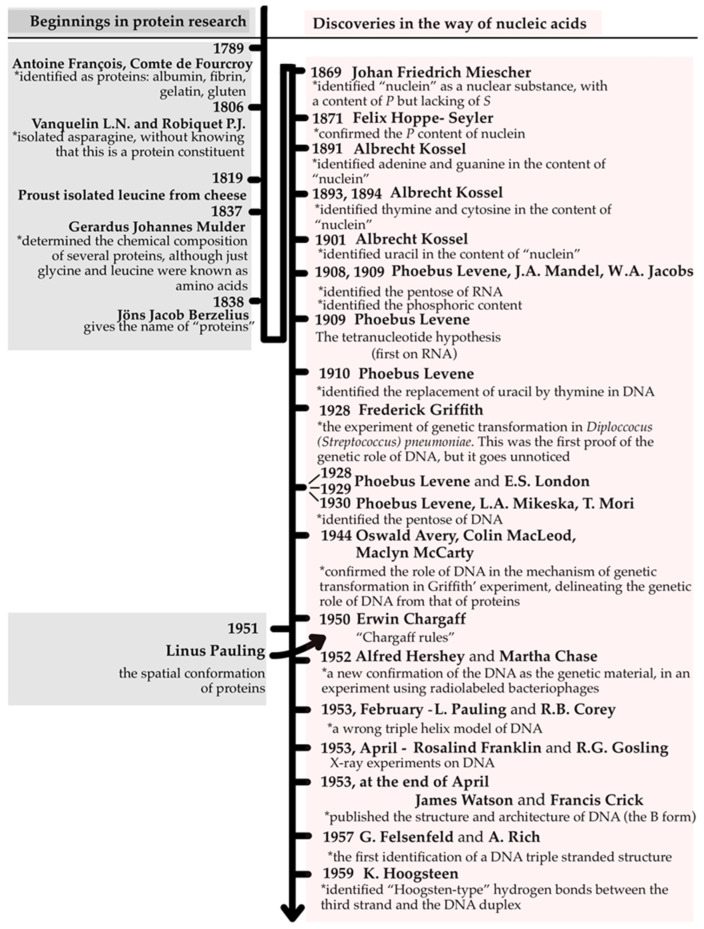
From Proteins to Nucleic Acids: Unveiling the Genetic Function and Chemical Structure of DNA (original).

This broader framework emerged from decades of scientific debate on the chemical nature of genetic material. During the late 19th and early 20th centuries, proteins and nucleic acids were studied largely as independent molecular entities, each with its own developmental trajectory. Their structural and chemical properties (discovered through laborious biochemical research) fueled an intense scientific controversy regarding which molecule carried the hereditary blueprint (Figure 2).

The subsequent subsections chronologically trace how proteins were initially favored as the informational substrate, how DNA gradually emerged as the correct candidate, and how structural discoveries, including triple and quadruplex DNA, have since reshaped our understanding of nucleic acid architecture and its functional diversity.

### 4.1. Before DNA: Proteins and the Quest to Understand Heredity

By the late 18th century, proteins were already recognized as key biological substances. In 1789, Antoine François de Fourcroy differentiated several protein types, including albumin, fibrin, gelatin, and gluten [75]. A major conceptual turning point occurred in the 1860s, when Johann Friedrich Miescher, working under Felix Hoppe-Seyler, identified a new acidic substance in cell nuclei (“nuclein”) that precipitated under acidic conditions and dissolved under alkaline conditions [76]. Although nuclein resembled proteins in certain properties, Miescher demonstrated that its protein-associated components were digested by pepsin, while a phosphorus-rich, sulfur-free fraction remained, distinct from known proteins (with phosphorus content later confirmed in 1871 by Hoppe-Seyler), indicating a unique elemental composition [77]. Miescher suspected that nuclein might play a role in heredity, especially after discovering that sperm heads contained nearly pure nuclein–protamine complexes (1872–1874) [76,78]. Nevertheless, he remained focused largely on pathological processes rather than heredity itself.

Between 1891 and 1901, Albrecht Kossel expanded on nuclein chemistry by identifying the nitrogenous bases (adenine and guanine, in 1891; thymine and cytosine, in 1893 and 1894; uracil, in 1901) [79]. Phoebus Levene, in the early 20th century, clarified the structural components of nucleotides, identifying the pentose in RNA [80], the replacement of uracil by thymine in DNA formula [81], the pentose in DNA [82,83], the presence of phosphoric acid being established as early as 1908–1909 [84,85]. Levene proposed the first structural model of nucleic acids: linear chains of nucleotides linked through phosphate groups. However, his Tetranucleotide Theory (1909) [86] incorrectly assumed equal quantities of the four bases in nucleic acids, leading many scientists to believe that nucleic acids were too simple to encode hereditary information.

In contrast, proteins were gaining increasing attention. Their structural diversity appeared far greater than that of nucleic acids, and the progressive identification of the 20 proteinogenic amino acids, from leucine, isolated by Proust from cheese in 1819 [87], to threonine, identified by William Cumming Rose in 1935 [88], further strengthened the perception that proteins were the most plausible candidates for encoding biological complexity.

By 1902, Franz Hofmeister and Emil Fischer, independently of each other, established that proteins are linear polymers of amino acids linked by peptide bonds, a discovery that fueled the belief that proteins, with their vast combinatorial potential, could serve as the molecular basis of heredity [75]. Proteins thus dominated early molecular thought, supported by the limitations of Levene’s model and by the increasing structural complexity uncovered in protein chemistry.

### 4.2. From Griffith to the Double Helix: The Birth of Molecular Genetics

The first experimental evidence suggesting that DNA could carry genetic information emerged in 1928, when Frederick Griffith discovered the phenomenon of bacterial trans-formation in *Streptococcus pneumoniae* [89]. However, his findings failed to alter the prevailing belief that nucleic acids lacked informational capacity. Only in 1944 did Oswald Avery, Colin MacLeod, and Maclyn McCarty identify DNA as the “transforming principle” [21], using carefully controlled biochemical experiments to eliminate proteins and other contaminants [90]. Their findings provided the first clear demonstration that DNA and not protein, is the hereditary material.

A decisive shift occurred in 1950, when Erwin Chargaff demonstrated that nucleo-tides do not occur in equal proportions, as Levene had proposed, but instead follow specific quantitative relationships (A = T and G = C) that implied structural complementarity [91]. This discovery discredited the Tetranucleotide hypothesis and opened the door for a new structural model.

In 1953, Watson and Crick proposed the DNA double-helix model [22], built upon (*i*) Chargaff’s base ratios [91], (*ii*) Rosalind Franklin and Raymond Gosling’s critical X-ray diffraction data that demonstrating the helical parameters of DNA [92], and (*iii*) the architectural inspiration of protein α-helices described by Linus Pauling and Robert Corey (1951) [93,94]. Simultaneously, in 1952, Alfred Hershey and his research assistant, Martha Chase, confirmed that bacteriophages inject DNA, and not protein, into bacterial cells during infection, providing a clean and elegant demonstration that DNA is the genetic material [95].

Undoubtedly, 1953 can be considered the year of DNA, when converging lines of evidence from biochemistry, microbiology, and structural biology firmly established DNA as the substrate of heredity, marking the birth of molecular genetics. The merits of Watson and Crick and that of Maurice Wilkins were crowned with the scientific recognition of the Nobel Prize in Physiology or Medicine in 1962. Rosalind Franklin died in 1958 when she was about 38, and because Nobel Prizes are not awarded posthumously, she was not eligible to receive a Nobel award for her critical work on the structure of DNA [96,97].

### 4.3. From Pauling’s Triple Helix to Real Triple and Quadruplex DNA

Although Pauling and Corey provided essential groundwork for understanding helical structures, their triple-helix model of DNA (1953) was incorrect [98]. They proposed a three-stranded DNA structure with phosphate groups located in the core and bases projecting outward, an arrangement incompatible with the molecule’s charge properties and with base-pairing rules. Nevertheless, their mistaken model proved historically important: it spurred Watson and Crick to articulate the correct double-helix structure, and it foreshadowed the existence of actual triple-stranded nucleic acid structures.

In 1957, Felsenfeld and Rich demonstrated stable triplex formation using synthetic polynucleotides (two poly-U strands with one poly-A strand) [99]. Hoogsteen later identified the alternative hydrogen-bonding interactions that stabilize triplexes (1959) [100], enabling T·A–T and C^+^·G–C base triplets [101,102]. These structures bind in the major groove of duplex DNA [103], influencing gene expression [104,105].

Even more striking was the discovery of G-quadruplexes, four-stranded DNA structures stabilized by stacks of G-quartets and metal cations (K^+^, Na^+^). Although predicted indirectly by the Swedish biochemist Ivar Bang in 1910 [106], when guanylic acid formed viscous gels [107,108,109], the structural basis of quadruplexes was elucidated by Martin Gellert and colleagues in 1962 [110]. Although they did not discover quadruplex DNA directly in chromosomes, they offered the first experimental evidence of specific molecular interaction (G-G Hoogsteen bond) that made the quadruplex model chemically and physically plausible [108,111]. By the early 1990s, G-quadruplexes were identified in telomeric DNA [112], with later studies showing that telomeric regions can form classical Watson–Crick duplex DNA, G-quadruplexes, and cytosine-rich *i*-motifs [113]. Of all these, the predominant structure is dictated by environmental conditions, such as pH and temperature, at their near-physiological values the Watson–Crick double helix being favored [114]. Rather than being mutually exclusive, these structures may coexist and interconvert within the telomeric region [115]. Recent findings proved that the non-canonical structures G-quadruplexes and *i*-motifs have a great biological significance, since they play crucial in vivo roles in maintaining the stability of telomeres, DNA replication, transcriptional regulation, alternative splicing, and translational regulation [111,116]. Moreover, their structural polymorphism is of great interest in new drugs and therapies development, particularly in cancer chemotherapy [117].

## 5. The Exception to the Rule: RNA Can Be the Substrate of Heredity!

The original formulation of the Central Dogma of Molecular Biology by Francis Crick in 1958 envisioned a strictly unidirectional flow of genetic information, from DNA to RNA to protein [23]. Although Crick later expanded this schema in 1970 to include additional, non-canonical information transfers, most notably RNA → DNA [24], his revisions were strongly influenced by earlier conceptual developments that had begun to erode the privileged status of DNA as the unique hereditary substrate.

One of the most visionary contributions in this regard came from Alex Rich, whose 1962 essay *On the Problems of Evolution and Biochemical Information Transfer* anticipated an RNA-centered view of heredity long before the formalization of the RNA World hypothesis [118]. Rich argued that RNA, by virtue of its dual capacity for information storage and catalytic activity, could have preceded DNA in evolutionary history and may still occupy a central role in biological information processing. He proposed that the earliest self-replicating systems were likely RNA-based, with DNA emerging later as a more stable repository, and proteins evolving as specialized functional molecules.

Although speculative at the time, and published more than a decade before the discovery of ribozymes, Rich’s reasoning introduced a conceptual shift: RNA was not merely an intermediate between DNA and protein, but a molecule capable of shaping, redirecting, and even initiating biological information flow [119]. This RNA-centric logic profoundly influenced subsequent challenges to the Central Dogma, including the discovery of reverse transcription and the recognition that viruses could use RNA as a hereditary substrate.

The studies on Rous sarcoma virus (RSV) provided the first direct experimental support for a non-DNA-based form of heredity, demonstrating that RNA could serve as the template for DNA synthesis through reverse transcription. Beginning with RSV’s identification by Peyton Rous in 1911 [120], and culminating in the detection of viral DNA intermediates and the discovery of reverse transcriptase in 1970 [25,26], these findings validated the type of alternative information pathways that Rich had conceptually anticipated.

### 5.1. The DNA Provirus Hypothesis and the Dawn of RNA → DNA Information Flow

In the early 1960s, it became increasingly clear that RSV replication could not be explained through the canonical DNA → RNA → protein pathway. RSV was shown to contain an RNA genome [121], yet small amounts of double-stranded DNA (*ds*DNA) were detected in infected cells [122]. This DNA was not part of the virion but rather a copy of the viral RNA, suggesting an unexpected flow of information, from RNA to DNA. This idea crystallized in the DNA Provirus Hypothesis, formulated conceptually between 1960 and 1961 and explicitly stated in 1964 by Howard M. Temin [123]. Temin proposed that the RNA genome of RSV must first be reverse-copied into DNA (the provirus), which then integrates into the host genome and directs the synthesis of progeny viral RNA.

At the time, this hypothesis contradicted the widely held belief that RNA viruses replicate exclusively via RNA → RNA and RNA → protein pathways, without DNA involvement. Indeed, Reich et al. had demonstrated in 1962 the RNA → RNA replication in other RNA viruses in the presence of actinomycin D, an antibiotic that inhibits DNA-directed processes, but not RNA-dependent ones [124]. Despite substantial skepticism, Temin persisted. He sought biochemical evidence of an RNA-dependent DNA polymerase in RSV-infected cells [125] and attempted to detect newly synthesized viral DNA labeled with 5-bromodeoxyuridine [126].

### 5.2. The Discovery of Reverse Transcriptase (1970)

A breakthrough occurred in 1967, when DNA-dependent RNA polymerase activity was identified in poxvirus virions (*ds*DNA viruses) [127,128], followed in 1968 by the discovery of RNA-directed RNA polymerase in reovirus virions (*ds*RNA viruses) [129,130]. These findings made it increasingly plausible that RSV might also contain a specialized polymerase.

In 1970, two independent groups, Temin and Mizutani [25], and David Baltimore [26], identified an RNA-dependent DNA polymerase, later named reverse transcriptase. This enzyme is inactivated by heat, loses activity when its RNA template is degraded by ribonuclease (as Temin and Mizutani demonstrated in 1970) [25], but remains resistant to actinomycin D [131].

Reverse transcriptase provided the long-sought molecular mechanism supporting the DNA Provirus Hypothesis. In 1975, Temin, Baltimore, and Renato Dulbecco were jointly awarded the Nobel Prize in Physiology or Medicine for this discovery [132].

### 5.3. Integration of the Provirus into the Host Genome

Following infection, reverse transcriptase synthesizes a complementary DNA strand (*c*DNA) from the viral RNA template, producing a transient RNA–DNA hybrid. Subsequent degradation of the RNA template by the polymerase’s ribonuclease activity permits synthesis of the second DNA strand. The result is a double-stranded DNA molecule, often circularized (as in RSV). Its integration into the host genome represents an essential subsequent step for viral replication and the stable inheritance of viral genetic information during host cell division, as elucidated by Temin and Mizutani (1970) [25], Baltimore (1970) [26], and Temin and Baltimore (1972) [133]. Additional enzymatic components, including RNA primers, ligases, and nucleases, assist in proviral DNA maturation and integration [134]. Comprehensive accounts of these processes were later provided by Bishop and Varmus [135,136].

### 5.4. Reverse Transcription, Recombination, and the Protovirus Theory

The replication of retroviruses involves frequent recombination, which can occur during reverse transcription, when the reverse transcriptase switches templates [137], or during proviral integration, when viral DNA recombines with cellular genomic sequences.

These recombination events are not random. They often occur at preferred genomic regions in host DNA, suggesting the existence of chromatin or sequence contexts that facilitate viral–host DNA exchange. Once integrated, the recombinant DNA (*r*DNA) containing viral inserts serves as a template for new viral RNA transcripts via host RNA polymerase, and these viral RNAs can again be reverse-transcribed into DNA, perpetuating cycles of re-combination, mutation, and reintegration. This iterative cycle was central to Temin’s Protovirus Theory (1971) [138], which proposed that retroviruses originate from normal cellular genetic components, reciprocal transfers of information between host DNA and viral RNA can generate variability, and repeated cycles of transcription, reverse transcription, integration, and recombination can lead to oncogenic mutations. In the same 1971 work, Temin also proposed a conceptual classification of oncogenic viruses and related genetic elements based on their mode of information transfer, explicitly distinguishing RNA tumor viruses that replicate via a DNA proviral intermediate from both DNA tumor viruses and RNA viruses lacking a DNA phase [138]. This framework established RNA → DNA → RNA replication as a defining biological category and provided an early functional classification of retroviruses prior to later molecular taxonomies.

Evidence accumulated over the 1970s expanded the scope of these ideas. Mizutani and Temin (1975–1976) identified both DNA and RNA polymerase activities in spleen necrosis virus virions, demonstrating unique features such as *de novo* synthesis of RNA primers required for reverse transcription [139,140]. Together, these discoveries overturned the classical view that RNA could not serve as a hereditary substrate. Reverse transcription and the provirus concept became foundational elements of modern molecular biology, retrovirology, and cancer genetics, marking one of the most profound expansions of the Central Dogma.

### 5.5. RNA-Centric Perspectives on Molecular Information Flow: From Rich’s Early Vision to Modern Evolutionary Implications

The development of molecular biology has often been portrayed as a progressive refinement of Francis Crick’s Central Dogma. Yet the emergence and consolidation of the *RNA World hypothesis* profoundly reshaped the theoretical foundations upon which the dogma was constructed, prompting a reassessment of the hierarchical relationship among DNA, RNA, and proteins. The RNA-centered perspective is not merely an evolutionary reconstruction of how life may have arisen. It now provides a powerful explanatory framework for the modern logic of gene regulation, cellular homeostasis, and molecular information transfer [141,142].

Historically, interpretations of the Central Dogma positioned DNA as the primary hereditary substrate. However, beginning in the 1950s, an alternative RNA-centered conception began to emerge. This perspective that now is crystallized as the *RNA World hypothesis*, was shaped significantly by the early and often under-recognized contributions of Alexander Rich, whose insights anticipated many of the discoveries that would later transform molecular biology.

The year 1953 is remembered for the elucidation of the DNA double helix, yet it also marked a conceptual turning point for RNA. Unlike DNA, RNA harbors an additional 2′-hydroxyl group, a feature later emphasized by Varshavsky for conferring enhanced chemical reactivity, structural flexibility, and branching potential, properties which are incompatible with long-term genetic stability, but ideal for catalytic and regulatory functions [143].

At the end of 1953, Rich and Watson began investigating whether RNA could adopt double-helical conformations. Using X-ray fiber diffraction, an approach that had successfully revealed DNA structure, they explored whether RNA obeyed similar organizational rules. Their 1954 published results [144,145] although inconclusive regarding RNA’s precise structure, revealed a crucial truth: RNA does not behave structurally like DNA, implying distinct biological roles beyond serving as a transcriptional intermediate. A decisive advance came in 1956, when Rich and David demonstrated that mixtures of synthetic polyribonucleotides [polyadenylic acid (poly A) and polyuridylic acid (poly U)] formed stable, complementary double helices [146]. The helical structure, with a diameter of ~26 Å for A-RNA (compared to ~20 Å for B-DNA), provided the first experimental demonstration that RNA alone can form a stable duplex through complementary base pairing. This discovery was historically transformative, as it satisfied one of the fundamental requirements of a hereditary system and expanded the conceptual framework for RNA’s biological potential [143,147].

Rich’s investigations in the early 1960s further reshaped the understanding of RNA function. He proposed that RNA may naturally occur in complementary pairs, with one strand acting as a messenger and the other functioning as an antisense regulatory molecule capable of modulating translation rates [148]. Rich described this complementary strand as a potential “control apparatus” that could turn protein synthesis on or off, anticipating, by decades, mechanisms later associated with antisense RNA, *micro*RNAs, *small interfering* RNAs, long noncoding RNAs, and RNA-guided epigenetic modifications. Long before RNA interference was formally discovered by Fire and Mello (1998) [47], Rich had already envisioned RNA as a central regulator of heredity, challenging the rigid hierarchy implied by the original formulation of the Central Dogma [149]. In 1962, Rich advanced a more radical conceptual step [118]. He proposed that early biological evolution may have been governed by RNA molecules possessing both informational and catalytic capacities, this being an early articulation of what would later be formalized as *the RNA World hypothesis*. His proposal anticipated subsequent formulations by Crick, Orgel, Woese, and ultimately Gilbert, establishing one of the earliest explicit arguments that RNA could serve simultaneously as genetic material and enzyme [119,150].

During the late 1960s, several researchers independently developed ideas consistent with an RNA-based primordial biology. For instance, Crick (1968) reasoned that early evolution must have relied on a simpler replicating entity predating the modern DNA–protein system, most plausibly RNA, which could combine templated replication with catalytic functions [151]. In parallel, Orgel (1968) emphasized RNA’s dual capacity as template and catalyst based on prebiotic chemistry, proposing RNA as a strong candidate for the earliest self-replicating molecule [152]. Woese (1967), in his foundational work on the genetic code, argued that transfer RNA served as a molecular fossil from a pre-protein era, suggesting that the complexity of modern translation emerged from simpler RNA-based mechanisms [153]. These converging perspectives culminated in Walter Gilbert’s influential 1986 proposal, which formally introduced the term *RNA World* [154]. Gilbert emphasized RNA’s dual informational and catalytic capacities and pointed to the ribosome’s peptidyl transferase center, composed entirely of *r*RNA, as compelling evidence that ancient life relied primarily on RNA catalysis [155,156]. The subsequent discovery of ribozymes provided direct experimental support, anchoring the *RNA World hypothesis* as a central model for understanding life’s origins [157].

The theoretical foundations laid by Rich, Crick, Orgel, Woese, and Gilbert were spectacularly validated in the early 1980s. Kruger et al. (1982) identified self-splicing activity in *Tetrahymena r*RNA, revealing the first natural ribozyme and establishing that RNA alone could mediate precise biochemical transformations [27]. Shortly thereafter, Guerrier-Takada and colleagues (1983) demonstrated that the RNA component of RNase P is the catalytic entity responsible for precursor *t*RNA processing [28] (*for details, see Section 9.4*). These discoveries established that RNA alone can mediate precise biochemical transformations. Modern structural biology has further shown that the ribosome is fundamentally a ribozyme, with the peptidyl transferase center composed entirely of *r*RNA. Work by Williams and colleagues positioned the ribosome as a molecular fossil of the RNA World, preserving ancient architectural signatures consistent with evolutionary descent from simpler RNA-based systems [158,159,160,161].

While the historical and mechanistic development of the *RNA World hypothesis* emerged from these conceptual and experimental advances, its plausibility ultimately depends on chemical feasibility. This dimension was elucidated by prebiotic chemistry, particularly the contributions of Dworkin, Miller, and Deamer [162,163], and later historical syntheses by Lazcano [164]. Experimental studies demonstrated that key RNA components, including ribose, nucleobases, activated ribonucleotides, and short catalytic oligomers, can form under geochemically plausible conditions. Dworkin et al. (2003) reviewed abiotic pathways enabling monomer formation, polymerization, and protocell assembly, suggesting models in which RNA catalysis and lipid vesicles co-evolved [163]. Lazcano (2016) traced the intellectual evolution of the *RNA World hypothesis*, showing how early speculative ideas gained coherence once ribozymes were discovered and prebiotic chemistry demonstrated that RNA was not only functional but chemically accessible [164].

A complementary modern perspective, articulated by Shapiro (2014) [165], proposes that RNA retains hierarchical primacy even in contemporary cells. In this view, DNA serves mainly as a stable archive, whereas RNA networks govern transcription, translation, chromatin architecture, genome stability, and epigenetic regulation [165]. This RNA-centric hierarchy reframes heredity as fundamentally RNA-directed, consistent with both early evolutionary logic and modern regulatory complexity.

Contemporary discoveries have validated Rich’s early intuition that complementary RNA strands can regulate gene expression. The regulatory architecture of modern cells, encompassing *micro*RNAs, *small interfering* RNAs, *piwi-interacting* RNAs, *long noncoding* RNAs, antisense transcripts, and circular RNAs (*see Section 9.3*), reveals RNA as a master regulator of genomic output. These findings complete a conceptual arc that began with Rich’s predictions in 1961, demonstrating that RNA is not merely a passive messenger but a central orchestrator of molecular information flow.

## 6. Ambiguities in Translation: Who Is the Messenger After All?

The transfer of genetic information from DNA to proteins through an RNA intermediate is the cornerstone of modern molecular biology. Although today this mechanism appears straightforward, DNA → mRNA → protein, the historical path toward identifying the true messenger was long, uncertain, and marked by conceptual confusion. Between the early structural proposals of DNA, the first experimental hints of RNA involvement in protein synthesis, and competing hypotheses assigning the messenger role to *r*RNA, *t*RNA, or even DNA itself, the identity of the molecule responsible for carrying genetic in-formation from nucleus to cytoplasm was far from evident.

### 6.1. Early Clues Linking RNA to Protein Synthesis

The publication of the DNA double-helix model by Watson and Crick in 1953 [22] provided a structural basis for heredity but did not clarify how genetic information is translated into proteins. In the same year, Watson and Crick detail the chemical structure of DNA molecule and describe its replication process, without bringing any argument about translation. Watson and Crick identify DNA as an essential constituent of chromosomes and, at the same time, the holder of the genetic information encoded at the level of genes. In the space defined by the winding of the paired polynucleotide chains, a polypeptide chain was described as being placed around the same helical axis, which had correctly assumed functions since that time, of controlling the coiling and uncoiling processes, as well as assisting in the packing of a polypeptide chain into its helical configuration [161].

In the decades leading up to these discoveries, several observations hinted at a relationship between RNA and protein synthesis. In 1947, Caspersson showed that cytoplasmic protein production correlates with increased levels of cytoplasmic RNA [166]. In the same year, André Boivin and Roger Vendrely proposed that RNA synthesis depends on DNA and that cytoplasmic proteins derive from these RNA molecules [167]. Jeener and Szafarz (1950) then suggested that RNA is synthesized in the nucleus and released into the cytoplasm, where it associates with large cytoplasmic particles before disappearing [168]. In retrospect, this was an early articulation of the concept of a messenger RNA exported to the cytoplasm for translation, even though the identity of the molecule remained unknown.

### 6.2. Phage Experiments and the First Glimpse of an Induced RNA

In 1953, Hershey reported that infection with bacteriophages induces the synthesis of a unique form of RNA, distinct from the normal bacterial RNA [169]. Volkin and Astrachan confirmed this in 1956, showing the transient production of “phage-induced RNA” from the host DNA template [170]. These findings hinted strongly at an intermediary RNA species, a conceptual precursor to messenger RNA, but this interpretation was not yet broadly accepted.

The uncertainty persisted in part because competing hypotheses were gaining trac-tion, including here a direct translation from DNA, proposed by Gamow (1954) [171], or a ribosomal RNA as the template for proteins, a view supported by the discovery of *r*RNA as a major ribosomal component [33,172].

### 6.3. Gamow, Crick, and the Search for a Decoding Mechanism

Gamow’s “key-and-lock” model (1954) proposed that amino acids interact directly with nucleotide triplets exposed in DNA grooves [171]. Although incorrect, this idea spurred significant interest in deciphering how nucleic acids encode proteins. In 1955, Crick’s letter to the “RNA Tie Club” refined this perspective by proposing the existence of an adapter molecule that matches amino acids to nucleic acid templates [173]. This visionary idea anticipated the discovery of *t*RNA. Between 1957 and 1960, Hoagland, Zamecnik, and colleagues demonstrated that soluble RNA (now known as *t*RNA) carries specific amino acids to ribosomes, each amino acid is attached to a specific cognate *t*RNA via an aminoacyl-*t*RNA synthetase, and ribosomes (identified by Palade in 1955, named by Roberts in 1958) are central participants in translation [33,34,174,175,176]. However, the presence of abundant *r*RNA in ribosomes led to confusion. Many researchers believed *r*RNA might serve as a template in protein synthesis or as a possible intermediary between DNA and proteins [172].

### 6.4. (1961): The Pivotal Year When the Messenger Was Finally Identified

The early 1960s were marked by intense debate regarding the identity of the true messenger. Matthaei and Nirenberg (March 1961) demonstrated that both ribosomal RNA and soluble RNA are required for protein synthesis, and they referred to *r*RNA as the possible “messenger” [177]. Only two months later, three landmark papers, by Jacob and Monod, Brenner et al., and Gros et al., finally clarified the existence and role of messenger RNA [178,179,180].

In their May 1961 review, François Jacob and Jacques Monod proposed that structural genes encode protein sequences, regulatory genes control protein synthesis via repressors and inducers, while the true messenger must be rapidly synthesized, short-lived, complementary to DNA, and able to associate with ribosomes [178]. Neither *r*RNA nor *t*RNA satisfied these criteria.

Jacob and Monod’s work on β-galactosidase induction in *E. coli* also suggested the presence of a transient intermediary that carries genetic information from DNA to the cytoplasm [181], a molecule they termed messenger RNA (originally abbreviated to M-RNA). The isolation of messenger RNA and the first unambiguous description of this molecule was announced in May 1961 by two publications in the Nature journal, initially by Brenner’s team [179] and later by Watson’s team [180] (Watson initially requested Brenner to withdraw an earlier publication in order to have time to verify the results).

### 6.5. The Translational Mechanism Becomes Complete

The identification of messenger RNA was an important breakthrough in finding a key component of the translational machinery. The dilemma of protein synthesis, initially on a DNA template [171], later on an RNA template, has now gained specificity with the identification of the messenger able of copying the genetic information from nuclear DNA and bringing it to the cytoplasmic level to be translated into polypeptide chains, with the participation of *t*RNA (soluble) and ribosomes.

Periods of scientific confusion have existed, these being governed by unclear or insufficiently elucidated hypotheses of the role of template *r*RNA in protein synthesis or of *t*RNA. None of these molecules met the expectations of the messenger molecule, including criteria such as its structure and life span. Once this molecule was identified, the translational picture became complete, by specifying the informational transfer from DNA to *m*RNA to proteins. Therefore, proteins represent the final result of such an informational flow, being demonstrated once again as structures that ensure the expression of heredity and not the substrate that stores and transmits the characters and traits of character in the offspring [182]. Thus, after more than a decade of debates, misinterpretations, and alternative hypotheses, the Central Dogma’s translation arm was firmly established: DNA → *m*RNA → protein.

## 7. Some Diseases Transmission by Means of Proteinaceous Agents Induces a New Confusion About Proteins: Are They the Expression of Heredity or Its Substrate?

The emergence of transmissible diseases caused by proteinaceous agents raised a fundamental question that challenged traditional views of heredity: can proteins act as substrates of inheritance, rather than merely its expression? This conceptual tension became particularly evident with the study of Transmissible Spongiform Encephalopathies (TSEs), diseases whose transmission appeared to occur without the involvement of nucleic acids. As early as 1967, J.S. Griffith proposed that a cellular protein could undergo a conformational conversion that propagates itself, giving rise to new pathological phenotypes [183].

This possibility created renewed ambiguity around the Central Dogma, since transmissible protein conformations seemed to defy the assumption that genetic information must be encoded only in nucleic acids.

### 7.1. Molecular Basis of Prion Diseases: Beyond the Early Paradox

Contemporary molecular evidence has clarified that the pathogenesis of TSEs does not represent a violation of the Central Dogma, but rather a distinct form of molecular pathophysiology with a strong genetic underpinning. Mutations and polymorphisms in the human *PRNP* gene (mouse *Prnp*) influence susceptibility, prion protein expression levels, and post-translational modification patterns, all of which alter the conformational stability of the native prion protein (PrP^C^) [184,185,186,187].

TSEs affect both humans and other mammals, having in common a process in which the normal α-helix–rich cellular prion protein PrP^C^ is converted into a β-sheet–rich, protease-resistant, aggregation-prone isoform PrP^Sc^ that finally acts as a template to convert more PrP^C^ molecules into the abnormal form [188,189,190]. This structural transition imparts remarkable biochemical stability, in the sense in which misfolded prion proteins resist heat, proteases, and chemical agents, promoting thus intracellular accumulation, plaque formation, and ultimately neurodegeneration [191].

### 7.2. Three Mechanisms of Etiology and a Strict Genetic Prerequisite

The etiopathogenesis of prion diseases classically knows three mechanisms, namely: (*i*) *acquired transmission* through contaminated food or iatrogenic routes; (*ii*) *inherited forms,* in which germline mutations in the prion protein gene predispose to spontaneous PrP misfolding; (*iii*) *sporadic forms*, where no clear environmental or genetic trigger is identified [187,192,193,194,195,196].

Crucially, experiments in *Prnp* knockout mice demonstrated that the presence of the PrP^C^ protein and, implicitly, the expression of its coding gene are essential conditions for the clinical expression of the disease and its transmission [186,187]. Without expression of the host-encoded prion protein PrP^C^), no infectious PrP^SC^ isoform can be generated or propagated. This was demonstrated directly in PrP-deficient animal models, which are completely resistant to prion infection, establishing that prions do not constitute an independent hereditary substrate but instead depend strictly on host gene expression [197,198,199]. Thus, although prion replication proceeds without a nucleic acid template, it remains genetically constrained by the availability of the PrP gene product, linking prion propagation to classical genomic inheritance rather than replacing it [200,201].

### 7.3. Infectivity, Transmission, and the Concept of Protein-Only Propagation

Horizontal prion transmission, such as the spread of scrapie among sheep and goats, initially suggested an infectious etiology. A key conceptual innovation was the idea of templated protein misfolding, now often described as an “epigenetic templated protein process” [193]. In this model, the incoming misfolded protein serves as a conformational seed that catalyzes the misfolding of endogenous PrP^C^.

Some scientists propose the term “transmissible” rather than “infectious” for prion diseases, especially when discussing iatrogenic transmission or experimentally induced forms [202]. However, the term “prion”, introduced by Stanley Prusiner in 1982 [29], explicitly emphasizes the original infectious nature of these particles: PROteinaceous INfectious particle (PROIN), shortened to “prion” for linguistic clarity [203].

Two major conceptual pillars underlie that a misfolded protein could act as a transmissible pathogenic agent independent of nucleic acids: (*i*) *Griffith’s protein-only hypothesis* (1967) and (*ii*) *Prusiner’s prion model* (1982). However, the idea of a proteinaceous etiological agent able of its own replication in the absence of instructions from a nucleic acid was clearly a radical extension of the Central Dogma as it was originally formulated by Crick in 1958. In 1997 Prusiner earned the Nobel Prize considering his research on prions. Undoubtedly, his work was laid on the “protein-only hypothesis” of J.S. Griffith, and on the first assumptions of a self-replicating protein (as the scrapie agent), and on a mechanism by which a protein could be infectious.

The first experimental evidence of the lack of nucleic acids in the structure of the scrapie agent was found in 1967 by researchers including T. Alper, J.S. Griffith, and D.C. Pattison [183,204,205]. They observed that scrapie infectivity was not destroyed by UV or ionizing radiation at levels that would typically inactivate nucleic acids in viruses or bacteria, as well as by heat and formaldehyde, two treatments that also inactivate most viruses [202,206,207]. Today, both the “protein-only hypothesis” as well as the “prion hypothesis” are accepted under the conditions of knowing a genetic determinism of the native protein and some genetic factors that increase its susceptibility to altered conformation. In this regard, a series of genetic factors, like polymorphisms in non-*PRNP* genes (other than the one responsible for prion protein synthesis), can influence the prion protein packaging (trafficking) by altering its post-translational modifications (PMTs). Affecting the enzymes involved in PMTs, processes like glycosylation (N-linked glycans attaching to specific protein sites) and GPI anchoring (a glycosylphosphatidyl—GPI anchor adding) are altered, leading to structural instability and aggregates forming in locations within and around cells, particularly in the brain, rather than remaining correctly on the cell surface [208,209,210].

The concept of prion-like propagation is also central to the understanding of protein misfolding in neurodegenerative diseases like Alzheimer’s, Parkinson’s, Huntington’s, and Amyotrophic Lateral Sclerosis (ALS). In these conditions, misfolded proteins, such as amyloid beta and tau in Alzheimer’s, alpha-synuclein in Parkinson’s, and huntingtin in Huntington’s, accumulate and can transfer from cell to cell. This process, similar to how prions function, involves these pathological seeds templating the misfolding of their normal counterparts, leading to the amplification and spread of the disease pathology throughout the brain [211,212,213].

These processes do not imply that such proteins serve as independent hereditary substrates; rather, they demonstrate a form of non-nucleic acid–based propagation of structural information, representing an *epigenetic-like transmission of conformational states* within tissues.

## 8. From Stable Structures to Jumping Genes in the Genome

The classical view of the gene as a fixed, stable, and immobile hereditary unit was fundamentally challenged in 1950, when Barbara McClintock discovered that specific DNA segments are capable of moving within the genome. These mobile sequences, later termed transposable elements (TEs) or transposons, revealed that genomes are intrinsically dynamic rather than static entities [30]. For this pioneering contribution, McClintock was awarded the National Medal of Science in 1970 and the Nobel Prize in Physiology or Medicine in 1983 [214].

Working with maize, McClintock described unusual kernel color patterns caused by DNA fragments that “jumped” into or near pigment genes, thereby altering their activity. When a transposon such as *Ds* (*Dissociation element*, located on the short arm of chromosome 9) inserts into the pigment gene on the short arm of chromosome 1, pigment synthesis is blocked, giving rise to colorless (yellow/white) spots. Insertion near, rather than within, the pigment gene disrupts its regulation and produces patchy or variegated coloration. When the transposon “jumps out”, pigment gene function can be restored, generating colored sectors on an otherwise colorless background [30,214,215,216,217].

The *Ds* element itself lacks the transposase gene required for movement and thus depends on the presence of the *Ac* (*Activator*) element elsewhere in the genome. *Ac* is an autonomous transposon whose position varies among maize lines. In the absence of *Ac*, *Ds* remains immobile and the genomic structure becomes stable again [217]. This maize system exemplifies “cut-and-paste” DNA transposons, which are mechanistically distinct from “copy-and-paste” RNA transposons (retrotransposons). Retrotransposons transpose via an RNA intermediate that is reverse-transcribed into DNA and inserted at a new genomic location, thereby increasing their copy number [218,219,220].

For several decades after their discovery, retrotransposons and other repetitive elements were largely interpreted as genomic “junk” or selfish DNA—byproducts of reverse-transcribed RNA reintegration with little or no contribution to organismal function [221,222]. This interpretation was supported by their high copy numbers, apparent lack of protein-coding capacity, and frequent association with insertional mutagenesis. Beginning in the late twentieth century, however, this view underwent a major conceptual shift. Accumulating evidence demonstrated that a subset of retrotransposon-derived sequences can be exapted into functional regulatory elements, including promoters, enhancers, splice sites, and non-coding RNAs [223]. In the human genome, *Alu* elements (~1.4 million copies) have been implicated in diverse regulatory contexts, ranging from transcriptional control and RNA editing to chromatin organization and stress-responsive gene regulation [165,224].

Early work by David Finnegan on retrotransposons in *Drosophila* was particularly influential, culminating in his 1989 proposal of a fundamental classification of eukaryotic transposable elements [225]. Regardless of their mechanistic differences, transposons have profoundly reshaped our understanding of genomic stability. They are now recognized as major drivers of genome evolution, modulators of gene expression, and powerful tools in biotechnology [226]. By changing genomic position or inserting new copies, transposons generate mutations that may increase genetic diversity and occasionally confer adaptive advantages, while also contributing to disease, aging, and cancer [227,228,229].

At the same time, it is increasingly recognized that the sheer abundance of retrotransposon insertions does not imply universal functionality. The vast majority of *Alu* and other retrotransposon copies are likely neutral or mildly deleterious, with only a small fraction being selectively retained through exaptation [223,230]. As emphasized by critical analyses, assigning function indiscriminately to repetitive elements risks conflating rare, context-dependent regulatory co-option with genome-wide adaptive purpose [66,231]. Retrotransposons should therefore be viewed primarily as sources of contingent genomic variation, from which occasional functional elements may emerge, rather than as uniformly functional components of the genome.

In this context, transposable elements do not merely introduce random mutations; they continuously reshape the architectural framework through which the DNA → RNA → protein flow is organized. By inserting regulatory sequences, rewiring promoters and enhancers, and creating or disrupting coding regions, mobile elements alter when, where, and how genetic information is transcribed and translated. Consequently, McClintock’s “jumping genes” contribute to transforming the Central Dogma from a static linear pathway into a historically contingent and genome-dependent process, in which information flow is persistently remodeled by mobile DNA. Importantly, these effects arise through stochastic insertion followed by selection or exaptation, rather than through directed or universally adaptive mechanisms [66,223,230,231].

## 9. From Fixed Pathways to Flexible Control: How Epigenetic and Epigenetic-like Factors Shaped the Central Dogma

Beyond DNA structure and a fixed linear DNA → RNA → protein flow, a wide set of *epigenetic* and *epigenetic-like* mechanisms reshape the functional implementation and biological consequences of information flow, not the fundamental directionality of information transfer itself. The term “epigenetics”, introduced in 1942 by Conrad Hal Waddington [232,233], refers to heritable changes in gene expression that do not involve alterations in the underlying DNA sequence, but nonetheless influence phenotype. These changes can be transmitted through mitotic inheritance (from a cell to its daughters) and, in some cases, through transgenerational inheritance across generations [234,235,236,237]. Classical epigenetic mechanisms act predominantly at the transcriptional level (DNA → RNA) via DNA methylation, histone modifications, nucleosome positioning, chromatin looping, and certain non-coding RNAs (*nc*RNAs). Other mechanisms function through feedback loops along the DNA → RNA → protein axis, involving transcription factors (TFs), histone acetyltransferases (HATs), and histone deacetylases (HDACs). By contrast, *epigenetic-like factors* do not necessarily produce stable chromatin marks, but modulate gene expression through non-canonical DNA conformations (A-DNA, Z-DNA), *nc*RNAs that regulate *m*RNA stability, translation or splicing, catalytic RNAs (ribozymes) acting post-transcriptionally.

### 9.1. Molecular Basis of Epigenetic Control in a Historical Framework: (i) DNA Methylation, (ii) Histone Modifications and Chromatin Remodeling, and (iii) Nucleosome Positioning

The classical view of gene regulation as a simple DNA → RNA → protein cascade has been transformed by the discovery of epigenetic mechanisms that finely tune when, where, and to what extent genes are expressed. Through DNA methylation, post-translational histone modifications (acetylation, methylation, phosphorylation, ubiquitylation), dynamic nucleosome positioning, and three-dimensional chromatin architecture, cells achieve a precise and reversible transcriptional control [233,238,239].

(*i*) *DNA methylation* was first described in 1948, when Rollin Hotchkiss identified 5-methylcytosine (5mC) in calf thymus DNA [31]. Only decades later did its central role in epigenetic regulation become clear. Foundational contributions by Holliday and Pugh [240] and by Compere and Palmiter [241] connected methylation patterns to gene regulation, differentiation, and developmental memory [242].

Mechanistically, DNA methylation involves the transfer of a methyl group (CH_3_) from S-adenosylmethionine (SAM) to the 5-carbon of the cytosine ring, catalyzed by DNA methyltransferases (DNMTs) [243]. Although methylation predominantly occurs at CpG dinucleotides, non-CpG methylation (CpA, CpT, CpC) is also observed in many organisms [244]. Environmental inputs, including diet, stress, and toxins, can profoundly reshape methylation landscapes, leading to long-lasting transcriptional changes [245,246].

While methylation leaves the DNA sequence unchanged, it promotes chromatin compaction, recruits repressive factors, and can sterically block transcription factor binding [234,242,247]. In plants, environmental stress frequently triggers demethylation of transposable elements, reactivating them and enabling new insertions that may create novel regulatory elements or disrupt existing genes [248,249,250].

(*ii*) *Histone modifications and chromatin remodeling.* In 1964, Allfrey, Mirsky, and Stern demonstrated that histones undergo acetylation and methylation and proposed that these modifications regulate RNA synthesis [32]. Because DNA is wrapped around histones to form nucleosomes, chemical changes to histones directly influence chromatin compaction and transcriptional output. Histone acetylation by HATs neutralizes positive charges on lysine residues, weakening histone–DNA interactions and generating open, transcriptionally active euchromatin. Conversely, HDACs remove acetyl groups, promoting chromatin condensation and repression [251,252]. Importantly, the enzymes responsible for all these modifications are themselves regulated transcriptionally, forming feedback loops that stabilize or reprogram transcriptional states across cell cycles [253].

(*iii*) *Nucleosome positioning.* Nucleosome positioning provides yet another layer of epigenetic regulation. In 1974, Kornberg first proposed that chromatin is organized into repeating subunits, nucleosomes [254]. This structural insight was followed by statistical models of nucleosome distribution [255] and by later work highlighting its essential role in transcriptional regulation [256]. Nucleosome positioning determines the accessibility of promoters, enhancers, and transcription factor binding sites. The arrangement of nucleosomes also governs higher-order chromatin folding and loop formation, bringing distal regulatory elements into contact with target promoters and thereby modulating transcription [257]. ATP-dependent chromatin remodeling complexes, recruited by transcription factors and non-coding RNAs, reposition, eject, or restructure nucleosomes, enabling rapid and reversible regulatory responses to developmental and environmental cues [258,259].

Together, DNA methylation, histone modifications, and nucleosome positioning transform gene expression from a simple consequence of DNA sequence into an environment-sensitive, reversible, and often heritable regulatory system. Within the framework of the Central Dogma, these mechanisms introduce a modulatory “filter” between DNA and RNA, such that a single genetic sequence can give rise to multiple transcriptional states depending on developmental context, metabolic signals, and environmental experience. This epigenetic plasticity has often been interpreted as providing a mechanistic substrate for neo-Lamarckian-like phenomena, whereby external conditions leave molecular “imprints” on transcriptional programs, imprints that may persist across cell divisions and, in some cases, across generations.

### 9.2. Non-Canonical A- and Z-DNA Forms and Their Epigenetic-like Influence on Gene Expression and Chromatin State

Besides chemical modifications, DNA can adopt alternative conformations most notably A-DNA and Z-DNA that modulate gene expression without altering the nucleotide sequence. These structural variants function as epigenetic-like regulators: they influence transcription by altering DNA accessibility, groove geometry, hydration, and protein-binding capacity, yet they do not necessarily generate heritable chromatin marks.

Under physiological conditions, DNA typically adopts the canonical right-handed B-form. However, transitions to A- or Z-DNA can occur in response to changes in ionic composition and cation concentrations (Na^+^, Mg^2+^, K^+^, Ca^2+^), hydration levels, specific sequence motifs, and DNA methylation patterns [260,261,262].

Two physicochemical parameters are particularly important for stabilizing the B-DNA conformation: balanced cation concentrations and adequate hydration of the DNA molecule. Monovalent and divalent cations bind the negatively charged phosphate backbone, neutralizing electrostatic repulsion and supporting the B-form helix [261,263,264]. When cation concentrations become excessively high, especially Mg^2+^ or other multivalent ions, over-neutralization promotes closer packing of phosphates on opposite strands, stabilizing the left-handed Z-DNA conformation, particularly in alternating purine–pyrimidine (GC-rich) sequences [262,265]. Hydration also plays a decisive role. B-DNA is stable under high humidity, with ~18–19 water molecules per nucleotide; this hydration shell supports its characteristic major and minor grooves and reduces electrostatic strain [266,267]. By contrast, reducing water availability favors transitions to A-DNA, which is less hydrated (~13–14 waters per nucleotide) [267]. Rosalind Franklin and Raymond Gosling first demonstrated the existence of two hydration-dependent DNA forms (A and B) in 1953 [49,268].

In B-DNA, both grooves are well defined, and the major groove provides a rich pattern of hydrogen-bond donors and acceptors that facilitates sequence-specific recognition by transcription factors and other regulatory proteins [269,270,271,272]. This structural “readout” makes B-DNA optimal for transcription initiation. A-DNA differs markedly: its minor groove is shallow and wide, while the major groove is deep and narrow, largely inaccessible to proteins and filled with structured networks of water and metal ions [269]. For this reason, A-DNA is generally incompatible with promoter recognition. Instead, the A-form is crucial during elongation, where the transient DNA–RNA hybrid within the transcription bubble adopts an A-like geometry. This geometry prevents steric clashes with the RNA 2′-OH group and stabilizes base pairing during transcript elongation (~8–10 nucleotides) [273,274].

Z-DNA, first observed in 1972 and structurally resolved in 1979 [50,51], exhibits a strikingly different topology: a left-handed helix with a zig-zag sugar-phosphate backbone, no conventional major groove, and a very narrow, deep minor groove [275]. Z-DNA is the least hydrated of the three major forms (~9 water molecules per nucleotide) [267], displaying distinct hydration patterns across its surface [275,276]. These biophysical properties restrict protein binding, yet specific Z-DNA-binding proteins (e.g., ADAR1-p150) recognize and stabilize this conformation, linking it to transcriptional regulation and innate immunity.

Interconversion between B-DNA and its alternative forms can be induced by dehydration or cation excess, but in vivo the major driving energy source is negative supercoiling generated during transcription [277,278]. Negative supercoiling behind the moving RNA polymerase facilitates B → Z transitions, especially in GC-rich promoters and enhancers where alternating dinucleotide steps favor Z-DNA formation [279]. Dinucleotide sequence also plays a crucial role. For instance, AA·TT steps (AT-rich regions) favor B-DNA, while non-alternating GC-rich clusters (e.g., consecutive GG·CC steps, as opposed to alternating CG repeats) tend to adopt A-DNA under reduced hydration. Alternating GC repeats (5′-CGCGCGCG-3′) have the highest Z-DNA potential, followed by CA/TG and TA steps (GC > CA > TA) [280,281,282,283,284,285].

Chemical modifications also influence transitions. CpG methylation enhances Z-DNA formation by increasing backbone rigidity and expelling water from the major groove [286,287]. Adenine deamination can similarly stabilize Z-DNA [262], while methylation-associated water exclusion subtly promotes A-DNA formation under certain conditions [288].

From the perspective of the Central Dogma, A- and Z-DNA demonstrate that genetic information is not encoded solely in the linear sequence of nucleotides, but also in higher-order conformational states that can be dynamically toggled by ionic milieu, hydration, supercoiling, protein binding, and methylation. These reversible structural polymorphisms act as epigenetic-like regulators, modulating transcriptional initiation and regulatory factor access without altering genotype. Thus, DNA structure itself becomes a regulatory variable, an additional physical layer of information flow that blurs the classical boundary between “genetic” and “epigenetic” regulation.

### 9.3. Non-Coding RNAs in Epigenetic and Epigenetic-like Regulation

Non-coding RNAs (*nc*RNAs) have emerged as central regulators of gene expression across multiple levels of biological information flow. Although only ~2% of human transcripts encode proteins, more than 70% of the genome is transcribed into *nc*RNAs, many of which exert regulatory functions [289]. Broadly, *nc*RNAs are classified into housekeeping *nc*RNAs (*t*RNA, *r*RNA, *sn*RNA, *sno*RNA) and regulatory *nc*RNAs (*mi*RNAs, *si*RNAs, *pi*RNAs, *lnc*RNAs) [290].

#### 9.3.1. Housekeeping Non-Coding RNAs: Constitutive Molecules with Epigenetic-like Effects

Although housekeeping *nc*RNAs primarily support basal cellular functions including translation, RNA processing, ribosomal assembly, they exert epigenetic-like regulatory effects by modulating RNA stability, translational fidelity, splice-site selection, and ribosome structure [291].

*t*RNA was the first *nc*RNA to be structurally characterized in detail, with Holley and colleagues reporting the sequence of *t*RNA^Ala^ in 1965 [35]. Although *t*RNA modifications are not classically heritable epigenetic marks, they strongly influence translation efficiency and fidelity. Modifications at positions 34 and 37 in the anticodon loop improve codon recognition, structural stability, and wobble pairing [292,293,294]. Pseudouridylation (Ψ), particularly in the D-loop, stabilizes *t*RNA structure and support accurate decoding [295].

Chemical modifications of *r*RNAs (methylation, pseudouridylation) affect ribosome architecture and translational dynamics, shaping global protein synthesis patterns [296].

*Small nuclear RNAs* (*sn*RNAs) (U1, U2 discovered in 1968 [36,37], followed by U4/U5/U6 [38]) are essential components of the spliceosome. They guide intron removal from pre-*m*RNAs. Differential *sn*RNA variants (e.g., U5D, U5E, U5F) contribute to alternative splicing, generating isoform diversity and thereby modulating gene expression in an epigenetic-like manner [297]. Mutations in *sn*RNA genes, including the neuron-specific U2 variant Rnu2–8, impair pre-*m*RNA splicing fidelity, causing transcriptome-wide missplicing that disproportionately impacts neuronal genes with complex intron–exon architectures and results in severe neurological phenotypes [298].

*Small nucleolar RNAs (snoRNAs),* first identified in 1970 [39], canonically guide site-specific 2′-O-methylation and pseudouridylation of ribosomal RNAs, thereby ensuring ribosome fidelity and translational accuracy [299]. Beyond these housekeeping functions, *sno*RNAs contribute to multiple layers of post-transcriptional regulation, including modulation of pre-*m*RNA splicing, stabilization of transcripts during nuclear export, and enhancement of *sn*RNA integrity and spliceosome precision through targeted RNA modifications [300,301,302]. Notably, a subset of *sno*RNAs encoded within imprinted genomic regions plays a direct role in human disease. In particular, clusters of C/D box *sno*RNAs located in the imprinted 15q11–q13 region, such as SNORD116 and SNORD115, are critically involved in Prader–Willi syndrome (PWS). Loss of paternal expression of these *sno*RNAs leads to severe neurodevelopmental and metabolic phenotypes, despite the absence of protein-coding gene disruption. This provides a compelling example of how non-coding RNAs can mediate heritable, parent-of-origin–specific regulatory effects without altering DNA sequence, functioning as bona fide epigenetic regulators rather than mere accessory RNAs [303,304,305].

#### 9.3.2. Regulatory Non-Coding RNAs: Direct Modifiers of Transcription and Translation

Regulatory RNAs (*nc*RNAs) include small *nc*RNAs, usually ranging from 20 to 30 nucleotides [small/short interfering RNAs (*si*RNAs), microRNAs (*mi*RNAs) and piwi-interacting RNAs (*pi*RNAs)] and long non-coding RNAs (*lnc*RNAs), which are typically more than 200 nt in length [289,290,306].

The first regulatory RNA discovered was micF in bacteria (1980s), which base-pairs with *ompF m*RNA to inhibit translation or promote decay in response to stress [307]. These prokaryotic *s*RNAs anticipate the eukaryotic *mi*RNA/*si*RNA pathways described in the 1990s [308].

The first major *lnc*RNA with a clear epigenetic function, X*ist*, was identified in 1991 [43,44]. X*ist* coats the X chromosome in cis and recruits ATRX and PRC2, inducing broad H3K27me3 deposition and transcriptional silencing [309]. *lnc*RNAs regulate gene expression through multiple mechanisms. At the transcriptional level, *lnc*RNAs can recruit or decoy chromatin remodeling complexes (like PRC2, LSD1), can participate at DNA methylation and histone modifications, altering thus DNA accessibility and influencing gene activation or repression, while some *lnc*RNAs can compete with TFs for binding sites on DNA (competitive inhibition) [310,311]. At the post-transcriptional level, through extended base-pairing, *lnc*RNAs can stabilize or promote translation, while partial base-pairing facilitates *m*RNA decay or inhibits its translation. Instead, in the absence of complementarity, *lnc*RNAs can suppress pre-*m*RNA splicing and translation by acting as decoys of RNA-binding proteins, preventing them from binding to their intended targets and thus altering gene expression [312]. HOX antisense intergenic RNA (HOTAIR) is a well-characterized *lnc*RNA that appears to be misregulated in a variety of cancers. *nc*RNA HOTAIR acts at both transcriptional and translational levels, as well as through other mechanisms like post-translational. By recruiting PRC2 via its 5′ end interaction with EZH2 (a subunit of PRC2), *lnc*RNA HOTAIR repress gene transcription through H3K27 trimethylase activity. In addition, HOTAIR interacts with lysine-specific demethylase 1A (LSD1), a histone modifier that suppresses transcription via H3K4 demethylation via its 3′ end [313]. Post-transcriptional, *lnc*RNA HOTAIR can act as a competing endogenous RNA (*ce*RNA), “sponging” up microRNAs (for example, *mi*R-148a) to upregulate the expression of their target genes (for example, of the EMT transcription factor Snail2) and to promote epithelial–mesenchymal transition (EMT) in esophageal carcinoma [314]. Post-translationally, it influences protein degradation through the ubiquitin-proteasome pathway [315,316].

*mi*RNAs were discovered in *C. elegans* in 1993 (lin-4) [45,46], a finding recognized with the 2024 Nobel Prize to Ambros and Ruvkun [317]. In contrast to *lnc*RNAs, which frequently act at both transcriptional and post-transcriptional levels, *mi*RNAs primarily function as post-transcriptional regulators. Canonically, *mi*RNAs bind to complementary sites within the 3′ untranslated regions (3′ UTRs) of target *m*RNAs, leading to translational repression and/or *m*RNA destabilization (*epigenetic-like factor*) [318,319,320]. *mi*RNAs can also influence transcription indirectly by targeting *m*RNAs encoding transcription factors or other regulatory proteins, thereby reshaping downstream gene expression networks without directly interacting with chromatin [321]. More rarely, a subset of *mi*RNAs has been reported to exert direct nuclear functions, where they associate with promoter regions or chromatin-associated RNAs and recruit chromatin-modifying complexes, resulting in transcriptional activation or repression. These promoter- or chromatin-associated *mi*RNA activities represent genuine epigenetic mechanisms but appear to be context-dependent and far less prevalent than canonical post-transcriptional regulation [322,323].

*si*RNAs are another type of non-coding RNA that usually act post-transcriptionally (similarly to *mi*RNAs, as epigenetic-like regulators), but under specific conditions they can also exert regulatory effects at the transcriptional level, functioning as *bona fide* epigenetic factors. It should be noted that transcriptional gene silencing is not an exclusive invention of RNA interference pathways. Earlier studies demonstrated that gene repression can occur through the act of transcription itself, independent of the functionality of the resulting RNA, a phenomenon broadly referred to as pervasive transcription or transcriptional interference [324]. Classical mechanisms such as promoter occlusion and transcriptional interference showed that transcription initiated from an upstream promoter can repress a downstream gene by altering chromatin structure or by polymerase interference, without requiring a functional RNA product [325,326]. In *Saccharomyces cerevisiae*, repression of the *SER3* gene by transcription of the upstream *SRG1* locus provided a clear demonstration that non-functional transcription can directly regulate gene expression through chromatin remodeling [327]. These findings established transcription-dependent silencing as a general regulatory principle that preceded and conceptually anticipated RNA-guided chromatin silencing mechanisms.

Within this broader framework, *si*RNA-mediated transcriptional gene silencing (TGS) represents a mechanistically refined pathway. By guiding Argonaute (Ago) proteins to specific gene promoters or chromatin-associated RNAs, *si*RNAs can initiate histone deacetylation, repressive histone methylation (such as H3K9), and DNA methylation, leading to stable transcriptional repression (TGS) [328,329,330,331,332]. At the post-transcriptional level, *si*RNAs guide (by using one of its strands) the RNA-induced silencing complex (RISC) to complementary *m*RNA sequences, resulting in endonucleolytic cleavage by the Ago protein and subsequent degradation by cellular exonucleases [333]. Fire and Mello’s discovery of RNA interference in *Caenorhabditis elegans* in 1998 demonstrated that double-stranded RNA (*ds*RNA) can trigger sequence-specific gene silencing [47], a finding recognized with the 2006 Nobel Prize in Physiology or Medicine. Although initially considered an exogenous defense mechanism, *si*RNAs are now known to arise endogenously from convergent transcription, sense–antisense transcript pairs, pseudogene-derived antisense RNAs, and hairpin RNAs (*hp*RNAs), underscoring their integration into normal gene regulatory networks [333].

*pi*RNAs were first identified in 2001 in *Drosophila* testes as small RNAs derived from the *Su(Ste)* locus, silencing Stellate transcripts to maintain male fertility [48]. The term *pi*RNA was later coined when these ~23–32 nt RNAs were found to associate with PIWI proteins (that belong to Ago proteins family) in both *Drosophila* and mice [48,334]. *pi*RNAs were originally found only in male and female reproductive tissues [335,336], their primary and best-understood function being to suppress transposon activity in germline cells. The mature *pi*RNA complex, including *pi*RNA-PIWI, is imported to the nucleus from the cytoplasm, where binds to complementary sites in nascent target of transposable elements transcripts, silencing their expression (*epigenetic factor*) [337]. *pi*RNA complexes can also silence transposable elements transcripts in the cytoplasm (*epigenetic-like factor*). The PIWI-Argonaute-Zwille (PAZ) domain of PIWI proteins recognizes and binds to the 2′-O-methylated 3′ end of *pi*RNA, while the MID domain of PIWI proteins binds to the 5′ uridine end, and the PIWI domain acts as an RNase H endonuclease to cleave the target transcript captured by *pi*RNA through complementary base pairing [338]. Because they maintain heritable TE repression across generations, *pi*RNAs function as epigenetic guardians of genome integrity [339,340].

Across all these classes, non-coding RNAs reshape the functional implementation and biological consequences of information flow, considering that housekeeping *nc*RNAs optimize translation and splicing (epigenetic-like), *lnc*RNAs reprogram chromatin, transcription, splicing, and translation, *mi*RNAs/*si*RNAs fine-tune *m*RNA networks and can modulate chromatin states, while *pi*RNAs enforce germline genome stability through epigenetic silencing of transposons. Together, they reposition RNA from a passive intermediary to the central regulatory node of the modern Central Dogma. Thus, DNA stores information, but RNA networks execute, sculpt, and modulate it in real time.

As anticipated conceptually long before their widespread experimental validation, non-coding RNAs can also function as molecular scaffolds and interaction brokers for proteins. As discussed by Brosius, RNA molecules are uniquely suited to mediate protein–protein interactions by binding multiple partners simultaneously, thereby facilitating functional or structural assemblies that might not otherwise occur through direct protein–protein contact alone [148]. In this context, RNA does not merely transmit regulatory signals but actively organizes molecular complexes. Moreover, ribonucleoprotein (RNP) assemblies can act as shuttles, directing associated proteins into specific subcellular compartments or microenvironments that individual components could not access independently due to the absence of appropriate targeting signals [341]. Such RNA-mediated scaffolding and transport functions further expand the regulatory repertoire of *nc*RNAs, reinforcing their role as dynamic organizers of intracellular architecture rather than passive regulatory intermediates.

### 9.4. Epigenetic-like Modifications and Functions of RNA: From Methylation to Catalysis

Beyond serving as intermediates in the flow of genetic information, RNAs themselves undergo extensive chemical modifications and can even possess intrinsic catalytic activity. These properties add multiple post-transcriptional regulatory layers that modulate RNA stability, processing, and translational output, functioning as epigenetic-like mechanisms that do not alter the underlying DNA sequence.

*RNA Methylation and Post-Transcriptional Regulation.* Among the >170 known RNA chemical modifications [342], N_6_-methyladenosine (m^6^A), 5-methylcytosine (m^5^C), N_1_-methyladenosine (m^1^A), and N_7_-methylguanosine (m^7^G) are some of the best characterized regulators of RNA fate. m^6^A is the most abundant internal modification in *m*RNA [343], first identified independently in 1974 by the laboratories of Desrosiers, Perry, and Kelly [344,345]. Its consensus sequence (G/A) (m^6^A) C was later defined by Schibler et al. [346] and Wei & Moss [347]. For instance, m^6^A influences gene expression by altering RNA–protein interactions, RNA structure, and ribosome dynamics. M6A-modified codons can reduce *t*RNA decoding accuracy, as m^6^A–U pairings are less stable than canonical A–U pairs [340,348]. In mammals, several RNA-binding proteins, including HNRNP family members, recognize m^6^A-marked transcripts and regulate splicing, processing, or stability [340]. The functional impact of m^6^A is position-dependent. Within coding sequences (CDS), for example, m^6^A can trigger CDS-m^6^A–dependent decay (CMD), contributing to ribosome pausing and transcript degradation [349], while within 3′UTRs, m^6^A promotes translation by recruiting selective reader proteins (YTHDF1/3, eIF3, METTL3) [350]. Other RNA species are also affected by methylation. m^1^A modifies *t*RNAs and *r*RNAs, altering their tertiary structure and modulating translational efficiency [348]. Ribosomal m^6^A marks, such as 18S m^6^A1832 and 28S m^6^A4220, stabilize ribosomal architecture and ensure proper subunit assembly [348]. Through such modifications, RNA molecules acquire regulatory features analogous to chromatin marks on DNA, positioning RNA methylation as a key epigenetic-like layer that fine-tunes information flow at the post-transcriptional level.

*Catalytic RNAs (Ribozymes) and the Functional Expansion of RNA.* Beyond chemical modification, some RNAs possess catalytic activity, acting as ribozymes capable of self-cleavage, ligation, or processing of other RNAs. Ribozymes are widespread across biological domains, including lower eukaryotes, bacteria, plants, viruses, and even vertebrates [351]. The first ribozymes, discovered independently by Cech and Altman in the early 1980s, were transformative for molecular biology. A self-splicing intron in *Tetrahymena* pre-*r*RNA (the ribosomal RNA precursor from *Tetrahymena thermophila*) is able of excising itself in vitro without protein assistance [27], and the RNA catalytic component of RNase P (M1 RNA) in *E. coli* processes pre-*t*RNAs autonomously [28]. These discoveries, recognized with the 1989 Nobel Prize in Chemistry, demonstrated unequivocally that RNA can function as both catalyst and information carrier, properties traditionally attributed to proteins and DNA, respectively.

However, RNA modifications and ribozymes together undermine the historical view of RNA as a passive messenger. Chemically modified bases act as dynamic regulatory annotations layered onto transcripts, while catalytic RNAs reveal that RNA molecules can perform mechanistic roles typically assigned to proteins. This dual capacity echoes the principles of the *RNA World hypothesis*: even in modern cells, RNA retains ancestral functionalities that merge storage, transformation, and regulation of genetic information. As such, RNA-based modifications and catalysis act as epigenetic-like mechanisms that enrich and modulate the canonical DNA → RNA → protein trajectory, confirming RNA’s unique position at the regulatory apex of the Central Dogma [342,352].

### 9.5. Cytoplasmic Inheritance and Its Influence on Nuclear Epigenetic Regulation

Cellular homeostasis relies on a constant bidirectional communication between mitochondria and the nucleus. Through anterograde signaling, nuclear genes control mitochondrial biogenesis and function, while through retrograde signaling, mitochondria transmit metabolic and stress-related cues back to the nucleus, reshaping chromatin states and gene expression programs [353]. This regulatory loop demonstrates that cytoplasmic factors can exert epigenetic influence on nuclear DNA.

Cytoplasmic (non-Mendelian) inheritance was first recognized in plants through the pioneering work of Correns and Baur on plastid transmission in *Mirabilis jalapa* and *Pelargonium zonale* [354]. In animals, mitochondrial inheritance was confirmed in 1974 in horse (*Equus caballus*)—donkey *(Equus asinus),* demonstrating predominantly maternal transmission of *mt*DNA [355]. *mt*DNA itself was first visualized by Margit and Sylvan Nass in 1963 [356] and fully sequenced in humans in 1981 [357]. Although generally maternally inherited, rare cases of paternal *mt*DNA leakage have been reported across species [358].

Mitoepigenetics encompasses chemical modifications of mitochondrial DNA, most notably 5-methylcytosine (5mC), first described in mouse *mt*DNA in the early 1980s by Pollack et al. [359]. Abnormal *mt*DNA methylation is linked to environmental exposures and disease states [360]. Because *mt*DNA lacks canonical histones, its packaging into nucleoids depends on TFAM (mitochondrial transcription factor A), whose phosphorylation and acetylation regulate its activity [361,362,363]. TFAM also participates in feedback to the nucleus by modulating NRF-1 (Nuclear Respiratory Factor-1) activity, thereby influencing its own expression [364]. Changes in TFAM levels or *mt*DNA copy number can indirectly reshape nuclear DNA methylation and transcriptional output [365,366].

Mitochondrial dysfunction triggers retrograde signals (via ROS accumulation, Ca^2+^ fluxes, NAD^+^ depletion) that reprogram nuclear epigenetic marks [367,368]. A central mechanism involves mitochondrial metabolites. For instance, α-ketoglutarate (α-KG), a cofactor for TET DNA demethylases and JmjC histone demethylases, links oxidative metabolism to DNA/histone demethylation [369,370]. Fumarate and succinate, when accumulated, inhibit α-KG–dependent dioxygenases, promoting hypermethylated chromatin [371]. In addition, NAD^+^ levels regulate sirtuin deacetylases (SIRTs, a class of HDACs, NAD^+^-dependent HDACs) and reduced NAD^+^ increases histone acetylation and opens chromatin [372,373]. Nevertheless, increased ROS resulted from improper mitochondrial translation further modulate nuclear epigenetics by affecting SAM availability, generating oxidative lesions (e.g., 8-hydroxy-2′-deoxyguanosine, 8-OHdG) that interfere with methylation, altering DNMTs, TET, HMTs (histone methyltransferases, enzymes that use SAM as a cofactor, like DNMTs), and HDMs (histone demethylases) activities, and reshaping histone acetylation patterns [374,375,376]. Thus, metabolic state becomes a direct determinant of chromatin structure.

Mitochondrial RNAs (mt-*r*RNAs, mt-*t*RNAs, mt-*m*RNAs) also undergo extensive modifications. For instance, adenine dimethylation in *r*RNAs was first suggested by Helser et al. in 1971 [377] and mapped by Poldermans et al. in the late 1970s [378,379]. Loss of these dimethylations impairs ribosomal subunit affinity and translational fidelity [380,381]. Mammalian mt-*r*RNAs possess fewer modifications than bacterial or cytoplasmic *r*RNAs, but the dimethylated residues m^6^_2_A936 and m^6^_2_A937 in 12S *r*RNA are essential for ribosome stability [382,383,384]. mt-*m*RNAs contain primarily m^1^A modifications, which generally repress translation by distorting Watson–Crick pairing in the coding sequence [385,386]. Li et al. (2017) showed that most m^1^A sites in mt-*m*RNAs localize to the coding region, contrasting with nuclear transcripts, where m^1^A enhances translation when located in the 5′ UTR [387].

Pseudouridylation (Ψ), catalyzed by PUS enzymes (pseudouridine synthases), stabilizes mt-*m*RNAs and mt-*r*RNAs and is essential for mitochondrial protein synthesis [388,389]. Defects in *t*RNA pseudouridylation cause MLASA syndrome (mitochondrial myopathy, lactic acidosis, and sideroblastic anemia), linking RNA modification to disease [390].

Both nuclear-encoded and mitochondria-encoded *nc*RNAs operate in mitochondrial translation and retrograde signaling. *p*iRNAs were detected in mitochondria in studies from 2012 and 2014, implicating mitochondria in *pi*RNA biogenesis and, indirectly, in DNA methylation and histone modification [353]. Evidence suggests mitochondria may host or store *mi*RNAs (mitomiRs—*mi*RNAs encoded by nuclear genes or mitochondrial genes), although canonical *mi*RNA biogenesis machinery is largely absent [353]. *lnc*RNAs are transcribed within mitochondria, some forming chimeric transcripts via trans-splicing, differing from nuclear *lnc*RNAs [353]. mt-*nc*RNAs can relay metabolic status to the nucleus, modulating nuclear gene expression.

Together, mitochondrial inheritance, mitoepigenetic marks, metabolite-dependent chromatin regulation, and mitochondria-derived *nc*RNAs demonstrate that nuclear gene expression is deeply shaped by cytoplasmic factors. These interactions extend the Central Dogma into a bidirectional information system, where metabolic environment and organelle function imprint regulatory states onto nuclear chromatin. This mechanistic framework provides a modern, experimentally grounded explanation for Lamarckian-like phenomena: environmental inputs and cellular experiences can leave persistent molecular signatures that influence, and sometimes transmit, patterns of gene expression across cell generations [60].

## 10. *De Novo* Gene Birth and Pseudogenization: Two Faces of the Same Genomic Dynamics

Advances in genomics over the past few decades have profoundly reshaped our understanding of genome plasticity. Contrary to the earlier view of DNA as a static repository of hereditary information, modern evidence reveals a molecular landscape in constant flux, where mutations, insertions, deletions, and structural rearrangements continuously remodel the repertoire of genetic functions. These processes directly affect the classical DNA → RNA → protein pathway, demonstrating that the flow of genetic information is itself evolutionary, historically contingent, and dynamically rewritten.

One of the most surprising insights to emerge from this new perspective is *de novo* gene birth. In this process, previously non-coding regions (intergenic DNA, introns, or remnants of transposable elements) gradually acquire the regulatory signals and structural features necessary for transcription and translation. Promoters and enhancers may evolve from random sequence fluctuations, open reading frames can arise through frameshift events or stop codon loss, and initially incidental transcription can be evolutionarily co-opted into functional expression [391,392,393]. What was once dismissed as “junk DNA” becomes the substrate for entirely new genes, a conceptual shift already anticipated by early analyses of retroposons as evolutionary seeds [394]. In this sense, *de novo* gene emergence expands the Central Dogma by showing that new DNA → RNA → protein pathways can be written into the genome over evolutionary time.

Although *de novo* gene birth was once considered exceedingly rare, systematic genome-wide surveys have revealed that it is far more common than previously assumed. Early clear examples were identified in *Drosophila* between 2006 and 2007, and subsequent analyses have shown similar patterns across many species (as reviewed in [393,395]). In humans, for instance, Wu et al. (2011) described approximately 60 protein-coding genes that arose *de novo* after divergence from the chimpanzee lineage [395]. These newly minted genes often encode short, structurally simple polypeptides at first, but may gradually integrate into cellular circuits, sometimes becoming essential components of regulatory or metabolic networks.

It should be emphasized, however, that only a small minority of sequence novelties generated by genomic turnover ultimately acquire biological function. In particular, many products of retrotranscription or sequence rearrangement give rise to retropseudogenes that are effectively “dead on arrival”, due to incomplete *c*DNA synthesis, disruptive mutations, or integration into genomic contexts lacking appropriate regulatory elements. Most such sequences remain transcriptionally silent or are rapidly inactivated, while only a small fraction escapes mutational decay and becomes evolutionarily stabilized through selection. Thus, *de novo* gene birth represents a probabilistic outcome within a much larger background of neutral or deleterious genomic experimentation [230,231,394].

*De novo* gene birth exists alongside a contrasting but equally pervasive process: pseudogenization, the inactivation of once-functional genes. Pseudogenes typically arise through mutations that introduce premature stop codons, frame shifts, or splice-site disruptions, or through the decay of promoter sequences that silence transcription. Once the DNA → RNA → protein flow collapses for a given locus, the gene becomes a non-functional remnant. The human genome contains between 10,000 and 20,000 such pseudogenes, numbers comparable to those of active protein-coding genes [396]. These silent genetic fossils preserve the evolutionary footprints of past physiological demands, ecological pressures, and metabolic shifts.

A well-known example is the repeated pseudogenization of the *GULO* gene, which encodes L-gulono-1,4-lactone oxidase, an enzyme required for the final step of vitamin C synthesis. This gene independently lost function in humans, other primates, guinea pigs, certain bat species, dogs, and several bird lineages. These species thus became reliant on dietary vitamin C. The recurrence of *GULO* pseudogenization across diverse lineages underscores how environmental abundance can relax selective pressures, allowing formerly essential genes to decay [397].

Although *de novo* gene birth and pseudogenization appear to be opposing processes, one generating new genes while the other eliminates old ones, they are fundamentally linked aspects of genome dynamics. Together, they illustrate innovation versus redundancy (some regions of the genome become novel sources of function, while others lose selective pressure and decay), shifts in selective landscapes (environmental or metabolic changes can trigger either adaptive emergence of new genes or the loss of genes no longer required), and plasticity of the information flow (the Central Dogma is not fixed but responds to genomic rewiring, allowing new DNA → RNA → protein pathways to arise or disappear). When considered together with epigenetic regulation, RNA-centered control, and cytoplasmic signaling, *de novo* gene birth and pseudogenization help transform the Central Dogma from a rigid linear model into a dynamic, historically contingent architecture of molecular heredity.

## 11. Conclusions

The historical and conceptual evolution of the Central Dogma of Molecular Biology illustrates the progression of biological thought from early philosophical reflections on heredity to a rigorously defined molecular framework. Foundational discoveries of the twentieth century, including the identification of DNA as the hereditary material, the elucidation of its double-helical structure, and Francis Crick’s formulation of the Central Dogma, established the core principles governing how genetic information is stored, transmitted, and expressed. Crick’s later refinement of the model, which acknowledged additional permissible modes of information transfer, anticipated the molecular complexity that subsequent decades of research would reveal.

Advances in genetics and molecular biology have since demonstrated that the canonical DNA → RNA → protein pathway operates within a highly dynamic and multilayered regulatory landscape. DNA methylation, histone modifications, chromatin organization, the activity of transposable elements, and the diverse functions of non-coding RNAs collectively shape transcriptional and post-transcriptional outcomes. In parallel, epitranscriptomic modifications, non-canonical DNA conformations such as A- and Z-DNA, protein-based templating phenomena exemplified by prions, and cytoplasmic–nuclear communication further emphasize that the execution of genetic information is profoundly context-dependent.

Importantly, these mechanisms do not invalidate the Central Dogma nor introduce alternative primary routes of informational flow. Instead, they refine its biological interpretation by revealing how canonical information transfer is modulated, constrained, and conditionally deployed. The Central Dogma thus remains a valid description of the directionality of molecular information, while its functional realization is shaped by regulatory layers that determine when, where, and to what extent genetic information is expressed.

From this perspective, heredity emerges not as a static genetic script but as a responsive and adaptive system. Genome dynamics, including *de novo* gene birth, diversification, and pseudogenization, further illustrate that the informational substrate itself is subject to continuous evolutionary remodeling. These processes highlight the historical contingency and plasticity of the DNA → RNA → protein framework, without challenging its fundamental logic.

By integrating historical insights with contemporary molecular discoveries, this review bridges classical models of heredity with modern regulatory and evolutionary concepts. The resulting framework preserves the conceptual integrity of the Central Dogma while embedding it within a broader molecular context that accounts for regulation, structural complexity, and genomic innovation. In doing so, it offers a more comprehensive and flexible understanding of how biological information is generated, interpreted, and reshaped across cellular and evolutionary timescales.

## Figures and Tables

**Table 1:** Heritable versus epigenetic-like mechanisms discussed in this review *.

Mechanism	Molecular Substrate	Germline/Transgenerational Heredity	Classification in This Review	Notes
DNA sequence variation	DNA	Yes	Genetic	Canonical hereditary substrate
DNA methylation (limited cases)	DNA	Context-dependent	Epigenetic	Stable inheritance only in specific systems
Histone modifications	Histones	Rare/context-dependent	Epigenetic	Often reset during gametogenesis
RNA modifications (m^6^A, m^1^A, Ψ)	RNA	No	Epigenetic-like	Dynamic, reversible, not germline transmitted
ncRNA regulation	RNA	Mostly no	Epigenetic-like	Regulatory, not autonomous inheritance
Cytoplasmic inheritance (mitochondria)	*mt*DNA	Yes	Genetic	Organelle genomes only
Cytoplasmic RNAs/proteins	RNA/protein	No	Epigenetic-like	Transient regulatory states
Prion propagation (mammals)	Protein	No	Epigenetic-like/pathological	Somatic templating only
Transposable elements	DNA	Yes	Genetic	Mobile but DNA-encoded
De novo gene birth	DNA	Yes	Genetic	New canonical information flow

* References for Table 1 are provided throughout the manuscript (see Section 7, Section 8, Section 9 and Section 10).

## Data Availability

This manuscript is a narrative integrative review based exclusively on previously published literature. No new datasets were generated or analyzed during the current study; therefore, data sharing is not applicable.

## Abbreviations and Key Molecular Terms

DNA	DeoxyriboNucleic Acid; the primary hereditary molecule storing genetic information
RNA	RiboNucleic Acid; mediates information transfer and regulatory functions between DNA and proteins
A, T, G, C, U	Adenine, Thymine, Guanine, Cytosine, Uracil; nucleotide bases
B-DNA	canonical right-handed DNA double helix predominant under physiological conditions
A-DNA	alternative right-handed DNA conformation favored by low hydration and certain ionic conditions
Z-DNA	left-handed DNA helix stabilized by high ionic strength, negative supercoiling, or CpG methylation
*n*DNA	nuclear DNA; the chromosomal DNA contained within the nucleus
*mt*DNA	mitochondrial DNA; the circular genome located within mitochondria
*c*DNA	complementary DNA; DNA synthesized from an RNA template by reverse transcription
*ds*DNA	double-stranded DNA; the canonical form of DNA composed of two complementary antiparallel nucleotide strands forming a double helix
*r*DNA	recombinant DNA; DNA molecules generated by combining genetic material from different sources, either naturally (e.g., through viral integration or transposition) or experimentally via genetic engineering
A-RNA	A-form RNA helix; the right-handed double-helical conformation adopted by RNA molecules and RNA-RNA hybrids
*ds*RNA	double-stranded RNA; an RNA molecule composed of two complementary RNA strands paired by Watson–Crick base pairing and typically adopting an A-form helical structure
*m*RNA	messenger RNA; a single-stranded RNA molecule transcribed from a DNA template
*t*RNA	transfer RNA; a small, structured RNA molecule that functions as an adaptor during protein synthesis
*r*RNA	ribosomal RNA; the structural and catalytic core of the ribosome
M-RNA	Messenger RNA (original abbreviation)
M1 RNA	the catalytic RNA component of bacterial RNase P
*nc*RNAs	non-coding RNAs; RNA molecules that are transcribed from the genome but do not encode proteins
*sn*RNAs	small nuclear RNAs; *nc*RNAs that are essential components of the spliceosome
*sno*RNAs	small nucleolar RNAs; *nc*RNAs that primarily localize to the nucleus and that are involved in the post-transcriptional modification and processing of ribosomal RNAs, as well as some *sn*RNAs and *t*RNAs
*mi*RNAs	microRNAs; short *nc*RNAs that regulate gene expression post-transcriptionally
*si*RNAs	small/short interfering RNAs; short double-stranded RNAs that mediate sequence-specific gene silencing through the RNA interference pathway
*pi*RNAs	PIWI-interacting RNAs; a class of small *nc*RNAs that associate specifically with PIWI proteins, a family of Argonaute proteins
*lnc*RNAs	long non-coding RNAs; a diverse class of RNA molecules longer than ~200 nucleotides that do not encode proteins
*s*RNAs	small (prokaryotic) non-coding RNAs; small *nc*RNAs predominantly found in prokaryotes that regulate gene expression at the post-transcriptional level
*hp*RNAs	hairpin RNAs; single-stranded RNA molecules that fold back on themselves to form a stem–loop (hairpin) structure due to internal base pairing, contributing to sequence-specific post-transcriptional gene silencing
*ce*RNAs	competing endogenous RNAs; RNA molecules, such as *m*RNAs, *lnc*RNAs, circular RNAs, pseudogene transcripts, that regulate gene expression by competing for shared *mi*RNAs
mt-*r*RNAs	mitochondrial ribosomal RNAs; ribosomal RNA molecules encoded by the mitochondrial genome and constituting the structural and catalytic core of mitochondrial ribosomes (mitoribosomes)
mt-*t*RNAs	mitochondrial transfer RNAs; transfer RNA molecules encoded by the mitochondrial genome and required for protein synthesis within mitochondria
mt-*m*RNAs	mitochondrial messenger RNAs; messenger RNA molecules transcribed from the mitochondrial genome and serve as templates for the synthesis of mitochondrially encoded proteins, primarily components of the oxidative phosphorylation system
mitomiRs	*mi*RNAs encoded by nuclear genes or mitochondrial genes
UTR	UnTranslated Region (of *m*RNA)
RSV	Rous sarcoma virus; an oncogenic retrovirus first identified in chickens, and that carries an RNA genome that is reverse-transcribed into DNA and integrated into the host genome as a provirus, providing key experimental support for extending the Central Dogma to include RNA → DNA information flow
TSEs	Transmissible Spongiform Encephalopathies; they are a group of fatal neurodegenerative disorders affecting humans and animals, exemplifying protein-based infectivity, where pathogenic prion conformations propagate by templating misfolding of the normal cellular prion protein, without involvement of nucleic acids
PrP^C^	Prion Protein, C—cellular form; this is the normal isoform of the prion protein, encoded by the *PRNP* gene and predominantly expressed on the cell surface where it is anchored to the plasma membrane via a GPI (glycosylphosphatidylinositol) moiety
PrP^Sc^	Prion Protein, Scrapie; this is the pathogenic misfolded isoform of the prion protein, derived from the normal cellular prion protein, characterized by a β-sheet-rich conformation, partial resistance to protease digestion, and a strong tendency to aggregate
*PRNP*	Prion Protein gene in humans; this is the gene encoding the human prion protein and that is located on chromosome 20 (20p13)
*Prnp*	Prion Protein gene in mouse; this is the ortholog of the human *PRNP* gene, located on chromosome 2
PROIN	Proteinaceous Infectious particle (transformed in PRION)
PMTs	Post-translational Modifications; these are covalent chemical modifications of proteins that occur after translation, altering protein structure, stability, localization, interaction capacity, and activity
GPI	GlycosylPhosphatidylInositol, a glycolipid anchor that covalently attaches certain proteins to the outer leaflet of the plasma membrane
ALS	Amyotrophic Lateral Sclerosis; this is a progressive neurodegenerative disease characterized by the selective degeneration of upper and lower motor neurons in the brain and spinal cord
TEs	Transposable Elements; they are mobile DNA sequences capable of changing their position within a genome
Ds	Dissociation element; this is a type of transposable element discovered in maize, known for its ability to move or “jump” within genome
Ac	Activator; this is an autonomous DNA transposable element
Alu	Alu elements; they are short interspersed nuclear elements that represent the most abundant transposable elements in the human genome
DNMTs	DNA MethylTransferases; they are a family of enzymes that catalyze DNA methylation
TFs	Transcription Factors; they are DNA-binding proteins that regulate gene expression by controlling the initiation, rate, and specificity of transcription by RNA polymerase
HATs	Histone AcetylTransferases; they are enzymes that add acetyl groups to specific lysine residues on histone proteins, primarily within histone tails
HDACs	Histone DeACetylases; they are enzymes that remove acetyl groups from lysine residues on histone tail, reversing the action of HATs
5mC	5-methylCytosine; this is a covalent DNA base modification in which a methyl group is added to the 5-carbon of cytosine, most commonly within CpG dinucleotides in eukaryotic genomes
SAM	S-Adenosyl Methionine; this is a universal biological methyl-group donor involved in a wide range of methylation reactions in cells
X*ist* RNA	X-inactive specific transcript RNA, a *lnc*RNA that plays a central role in X-chromosome inactivation in female mammals
ATRX protein	Alpha-Thalassemia mental Retardation X-linked protein; this is a chromatin-remodeling protein encoded by *ATRX* gene on the X chromosome
PRC2	Polycomb Repressive Complex 2; this is a multisubunit epigenetic regulator that mediates transcriptional repression through histone modification
H3K27	the 27th amino acid in Histone H3, which as a lysine (written as “K”)
HOX	HomeobOX; HOX genes are a highly conserved family of transcription factor-encoding genes that control anterior–posterior body patterning and cell identity during development
HOTAIR	HOX Transcript Antisense Intergenic RNA; this is a well-characterized *lnc*RNA that functions as a chromatin-associated regulatory RNA, playing a key role in epigenetic gene silencing
EZH2	Enhancer of Zeste Homolog 2; this is a histone methyltransferase and the catalytic core subunit of Polycomb Repressive Complex 2 (PRC2), playing a central role in epigenetic gene silencing
LSD1	Lysine-Specific Demethylase 1A; this is a histone demethylase that plays a key role in epigenetic regulation of gene expression by removing methyl groups from specific lysine residues on histone tails
H3K4	the fourth amino acid in Histone H3, which is a lysine
EMT	Epithelial–Mesenchymal Transition (process); this is a biological process in which epithelial cells lose their cell–cell adhesion and polarity and acquire mesenchymal characteristics, including enhanced migratory and invasive capacity
ESCC	Esophageal Squamous Cell Carcinoma
Ago	Argonaute; this is a class of highly conserved family of RNA-binding proteins that are central components of RNA-mediated gene silencing pathways
H3K9	the ninth amino acid in Histone H3, which is a lysine
TGS	Transcriptional Gene Silencing; this is a regulatory mechanism in which gene expression is repressed at the level of transcription, typically through chromatin modification, rather than by degradation of *m*RNA after it is produced
RISC	RNA-Induced Silencing Complex; this is a multiprotein effector complex that mediates post-transcriptional gene silencing (PTGS) by using small RNAs as a sequence-specific guides to target complementary RNA molecules
*Su(Ste)*	*Su(Ste)* locus, a Y chromosome linked repetitive locus in Drosophila melanogaster that produces *pi*RNAs complementary to Stellate (Ste) sequences located on the X chromosome
PIWI	P-element-Induced Wimpy testis; a subfamily of Argonaute proteins that specifically binds *pi*RNAs (PIWI-interacting RNAs) and that is essential for germline development and fertility
PAZ	PIWI-Argonaute-Zwille; this is a conserved RNA-binding domain found in Argonaute and PIWI proteins that specifically binds the 3` end of small RNAs (*mi*RNAs, *si*RNAs, *pi*RNAs), typically recognizing the two-nucleotide 3` overhang
m^6^A	N_6_-methylAdenosine; this is a post-transcriptional RNA modification in which a methyl group is added to the nitrogen-6 position of adenosine
m^5^C	5-methylCytosine; this is a post-transcriptional RNA modification in which a methyl group is added to the carbon-5 position of cytidine
m^1^A	N_1_-methylAdenosine; this is a post-transcriptional RNA modification in which a methyl group is added to the nitrogen-1 position of adenosine
m^7^G	N_7_-methylGuanosine; this is a post-transcriptional RNA modification in which a methyl group is added to the nitrogen-7 position of guanosine
HNRNP	Heterogeneous Nuclear RiboNucleoProtein; this is a large family of RNA-binding proteins that associate with nascent and processed RNAs, primarily in the nucleus, forming ribonucleoprotein (RNP) complexes
CDS	Coding Sequence; this is the portion of a gene or mRNA that is translated into protein
CMD	CDS-m6A Decay; this is a translation-coupled mRNA decay pathway triggered by N6-methyladenosine modifications within the coding sequence
YTHDF	YTH Domain Family; this is a family of cytoplasmic m^6^A reader proteins that recognize N6-methyladenosine (m^6^A) modifications on RNA and regulate *m*RNA fate
eIF	eukaryotic Initiation Factor; this is a group of proteins that regulate the initiation phase of protein synthesis in eukaryotic cells by controlling ribosome recruitment to *m*RNA
METTL	MethylTransferase-Like protein; this is a family of enzymes that catalyze RNA methylation, most prominently N6-methyladenosine (m6A), thereby regulating RNA metabolism and gene expression
ROS	Reactive Oxygen Species; these are chemically reactive oxygen-derived molecules that arise as natural by-products of cellular metabolism and function both as signaling molecules and sources of oxidative stress
NAD^+^	Nicotinamide Adenine Dinucleotide; this is a ubiquitous redox cofactor and metabolic signaling molecule essential for cellular energy metabolism, epigenetic regulation, and stress response
TFAM	Mitochondrial Transcription Factor A; this is a nuclear-encoded DNA-binding protein essential for mitochondrial DNA maintenance, transcription, and packaging
NRF-1	Nuclear Respiratory Factor 1; this is a nuclear transcription factor that coordinates the expression of genes required for mitochondrial biogenesis, respiration, and mitochondrial-nuclear communication
α-KG	Alpha-KetoGlutarate; this is a central metabolic intermediate of the tricarboxylic acid cycle that also functions as a key regulator of epigenetic and epigenetic-like processes
TET	Ten-Eleven Translocation (enzymes); this is a family of α-ketoglutarate-dependent dioxygenases that catalyze active DNA demethylation and play a central role in epigenetic regulation
JmjC	Jumonji C (protein domain); this is a conserved protein domain found in histone demethylases that catalyzes the removal of methyl groups from histone lysine residues, playing a central role in chromatin dynamics and epigenetic regulation
SIRTs	Sirtuins (enzymes); this is a conserved family of NAD^+^–dependent deacetylases and ADP-ribosyltransferases that regulate chromatin structure, gene expression, metabolism, stress responses, and aging
8-OHdG	8-hydroxy-2′-deoxyGuanosine; this is a widely used biomarker of oxidative DNA damage resulting from the oxidation of the guanine base in DNA
HMTs	Histone MethylTransferases; this is a class of enzymes that catalyze the transfer of methyl groups to specific lysine or arginine residues on histone proteins, thereby regulating chromatin structure and gene expression
HDMs	Histone DeMethylases; this is a class of enzymes that remove methyl groups from methylated histone residues, thereby reversing histone methylation marks and dynamically regulating chromatin structure and gene expression
m^6^_2_A	Dimethylation of Adenosine residues at the N_6_-position
Ψ	Pseudouridine; this is the most abundant post-transcriptional RNA modification formed by isomerization of uridine, in which the uracil base is linked to ribose via carbon-carbon bond instead of the usual nitrogen-carbon bond
PUSs	PseudoUridine Synthases; this is a family of RNA-modifying enzymes that catalyze the site-specific conversion of uridine to pseudouridine in RNA molecules
RPUSD3, RPUSD4	RNA PseudoUridine Synthase D3, D4; these are mitochondrial pseudouridine synthases involved in post-transcriptional modification of mitochondrial RNAs
MLASA	mitochondrial Myopathy, Lactic Acidosis, and Sideroblastic Anemia
*GULO*	*L-gulonolactone oxidase* (gene)
